# Waist circumference and grip strength and their joint relations to type 2 diabetes incidence in UK Biobank

**DOI:** 10.1186/s12916-026-04907-8

**Published:** 2026-05-07

**Authors:** Johanna Wirler, Michael J. Stein, Hansjörg Baurecht, Patricia Bohmann, Heinz Freisling, Claas Lendt, Anja M. Sedlmeier, Michael F. Leitzmann

**Affiliations:** 1 Department of Epidemiology and Preventive Medicine, University of Regensburg, 93053 Regensburg, Germany; 2 Institute of Epidemiology, Helmholtz Zentrum München, German Research Center for Environmental Health (GmbH), 85764 Neuherberg, Germany; 3 Department of Epidemiology and Preventive Medicine, Medical Sociology, University of Regensburg, 93053 Regensburg, Germany; 4 International Agency for Research on Cancer (IARC-WHO), 69366 Lyon, France; 5 Center for Translational Oncology, University Hospital Regensburg, 93053 Regensburg, Germany; 6 Bavarian Cancer Research Center (BZKF), 93053 Regensburg, Germany

**Keywords:** Type 2 diabetes, Waist circumference, Grip strength, Dynapenic abdominal obesity

## Abstract

**Background:**

Waist circumference and grip strength are each associated with type 2 diabetes (T2D) risk, but their joint associations have been less well studied.

**Methods:**

We examined the separate and joint associations of waist circumference and grip strength with incident T2D among 483,578 adults aged 40–69 years (55% women) without T2D at baseline (2006–2010) from UK Biobank. Waist circumference was measured by trained staff and categorized using World Health Organization thresholds. Grip strength was assessed using a hydraulic dynamometer and categorized into age- and sex-specific tertiles. Incident T2D was ascertained through linkage to hospital inpatient records until 2022. Hazard ratios (HRs) and 95% confidence intervals (CIs) were estimated using Cox proportional hazards regression, adjusting for sociodemographic, lifestyle, and clinical covariates.

**Results:**

During 13.0 years of follow-up (6.3 million person-years), 30,240 participants (6.3%) developed T2D. Compared to individuals with low waist circumference (men: ≤ 94 cm, women: ≤ 80 cm), HRs were 2.11 (95% CI 2.03–2.19) for those with intermediate (men: 95–102 cm, women: 81–88 cm) and 5.48 (95% CI 5.30–5.66) for those with high waist circumference (men: > 102 cm, women: > 88 cm). Compared to individuals with high grip strength, HRs were 1.08 (95% CI 1.05–1.11) for those with intermediate and 1.35 (95% CI 1.32–1.39) for those with low grip strength. Joint analyses showed the highest risk among participants with the combination of high waist circumference and low grip strength (HR 7.68, 95% CI 7.22–8.17) compared to individuals with the combination of low waist circumference and high grip strength. Associations between waist circumference and T2D were stronger in women, whereas associations with grip strength were stronger in men. Both patterns were more pronounced among younger adults.

**Conclusions:**

Waist circumference and grip strength were separately and jointly associated with T2D risk. The combination of high waist circumference and low grip strength conferred the greatest risk. Joint assessment of waist circumference and grip strength identifies individuals at particularly elevated risk and may inform preventive strategies, though formal evaluation of incremental predictive utility is needed.

## Background

Type 2 diabetes (T2D) is one of the fastest-growing public health challenges worldwide, contributing to cardiovascular disease, disability, and premature mortality [1, 2]. Approximately 589 million adults currently live with diabetes, corresponding to one in nine adults, and an estimated 252 million cases remain undiagnosed. The burden of T2D continues to rise, driven by aging populations, increasingly sedentary lifestyles, and the growing prevalence of obesity, and is projected to reach 853 million by 2050 [1, 3].

Among the various anthropometric indicators, waist circumference has emerged as a particularly strong predictor of T2D risk [4–6]. It reflects abdominal obesity, which is closely linked to insulin resistance and metabolic dysfunction, key mechanisms underlying T2D pathogenesis [7]. Longitudinal studies have shown that increases in waist circumference over time are associated with a higher risk of developing T2D [8, 9], emphasizing the central role of abdominal obesity in diabetes development.

In parallel, muscular fitness – encompassing muscular strength and muscular endurance and often assessed by handgrip strength – has gained attention as a potential protective factor against T2D [10–13]. Data from UK Biobank showed that lower grip strength was associated with a higher risk of T2D, after accounting for general or central adiposity and other established T2D risk factors [14], suggesting that this simple and low-cost measure may serve as a practical complement to adiposity measures in clinical risk assessment [15].

Despite the growing body of evidence on the separate roles of abdominal obesity and muscular fitness, their combined influence on T2D risk remains underexplored. Previous studies have mainly focused on general obesity and cardiorespiratory fitness, the latter referring to the capacity of the cardiovascular and respiratory systems to supply oxygen to the body during physical activity and is typically assessed by maximal or submaximal exercise testing, such as treadmill or cycle ergometry [16, 17]. Emerging population-based evidence suggests that individuals with concurrent abdominal obesity and low muscular strength may have particularly elevated diabetes risk, though prospective data integrating abdominal obesity with handgrip strength are limited to a single Asian study which reported inconclusive results for the joint association [18]. No prior study has examined this joint association in a European population. To address this research gap, the present study investigated the separate and joint associations of waist circumference and grip strength with incident T2D in a large cohort of adults in UK Biobank.

## Methods

### Study population and data collection

UK Biobank is a prospective cohort that recruited over 500,000 UK participants aged 40-69 years at baseline (2006-2010). The study collected sociodemographic, lifestyle and clinical information. Assessments included touchscreen questionnaires, interviews, physical and functional measurements, and biological samples [19]. We excluded participants with prevalent T2D – any individual with hospital inpatient records of T2D on or before baseline – and those with missing values for any exposure or outcome variable. To mitigate potential reverse causation, we excluded T2D cases that occurred within the first two years of follow-up, resulting in a final study population of 483,578 individuals (Additional file 1: Figure S1). UK Biobank received ethical approval as a data and tissue repository for research purposes from the Northwest Multi-Centre Research Ethics Committee. Written informed consent was obtained from all participants prior to data collection [19].

### Assessment of waist circumference and grip strength

During the baseline visit, clinical staff measured waist circumference in centimeters using a Seca 200cm tape measure at the smallest part of the trunk, or the belly button, during exhalation [20]. Waist circumference was categorized according to World Health Organization sex-specific thresholds into three waist circumference groups: substantially increased risk (high waist circumference: > 102 cm for men (M), > 88 cm for women (W)), increased risk (intermediate waist circumference): 95–102 cm (M), 81–88 cm (W); no risk (low waist circumference): ≤ 94 cm (M), ≤ 80 cm (W) [21]. Grip strength was assessed using a Jamar hydraulic hand dynamometer following a standardized protocol. Participants performed two maximal grip strength attempts per hand, and the highest value was retained [22]. Given that UK Biobank participants tend to be healthier than the general population, we used age- and sex-specific tertiles to categorize grip strength into “low”, “intermediate” and “high” to better reflect the distribution within this cohort rather than applying recently-proposed reference values (Additional file 1: Table S1) [23].

### Type 2 diabetes ascertainment and cohort follow-up

Participants’ vital status was determined through linkage with routine healthcare data and national death registries [24]. Follow-up began at the assessment date and ended at the date of T2D diagnosis, date of complete follow-up (October 2022 for England, August 2022 for Scotland and May 2022 for Wales) [25], loss to follow-up, or date of death, whichever occurred first. The endpoint was incident T2D, identified through linkage with hospital inpatient records of ICD-10 code E11 or equivalent ICD-9 codes (e.g., 250.00), with the incident date assigned as the first diagnosis, regardless of other concurrent conditions [19]. Completeness of follow-up was ensured by additional linkage with national health registries, which provide near-complete case ascertainment and accurate survival time. Furthermore, estimates for established risk factors are consistent across data sources, supporting the validity of using hospital records for diabetes case ascertainment within UK Biobank [26].

### Covariates

Potential confounding covariates were determined using evidence-based directed acyclic graphs (DAGs), with details in Additional file 1: Figure S2 [27]. DAGs were used to visualize causal structures and to identify confounding variables while distinguishing them from intermediate variables or colliders [28]. Briefly, we stratified by sex, age, and study region, and adjusted for smoking, alcohol use, socio-economic status, education, sedentary behavior, and healthy diet score (Additional file 1: Table S2 [29, 30]). In sensitivity analyses, we additionally adjusted for physical activity volume, height, and family history of diabetes to evaluate the robustness of the results with respect to further potential confounding.

### Statistical analysis

Descriptive analyses included absolute and relative frequencies for categorical variables, as well as mean values and standard deviations for continuous variables. Age standardization was performed using inverse probability weighting, based on a uniform reference distribution across age groups, to reduce confounding by age. We performed Cox proportional hazards regression using age as the underlying time scale [31] to estimate hazard ratios (HRs) and corresponding 95% confidence intervals (CIs) for waist circumference and grip strength in mutually adjusted models. We investigated their combined relations by modeling a nine-level joint exposure variable, representing all possible combinations of waist circumference categories and grip strength tertiles. Participants who had a low waist circumference and a high grip strength served as the reference group, based on the assumption that this combination represents the lowest risk profile for incident T2D [32]. The proportional hazards assumption was evaluated using Schoenfeld residuals, including global and covariate-specific tests, complemented by visual inspection of scaled Schoenfeld residual plots. We tested for multiplicative interaction by generating cross-product terms between waist circumference and grip strength, the statistical significance of which were tested using a Likelihood ratio test. To assess whether joint associations exceeded the sum of individual associations, we additionally evaluated additive interaction using the relative excess risk due to interaction (RERI), the attributable proportion (AP), and the synergy index (S) [33–35]. Measures of additive interaction were calculated based on HRs derived from Cox proportional hazards models with a common reference group. CIs were estimated using the delta method based on the variance-covariance matrix of the regression coefficients. Further analyses were stratified by sex and by age (< 60 years, ≥ 60 years) to assess patterns across major characteristics, with 60 years chosen as a cut-off because it broadly distinguishes midlife from older adulthood and corresponds to a clinically meaningful age threshold for T2D risk [1].

In additional analyses, we evaluated age- and sex-specific cut-off values for normalized grip strength (absolute grip strength in kilograms divided by height in meters squared). This approach, supported by recent international normative data, provides a size-adjusted indicator of muscular strength that facilitates valid comparisons across age, sex, and populations [23]. Furthermore, to assess the robustness of our grip strength categorization, we applied normative reference values for absolute grip strength proposed by Tomkinson et al. [23], classifying participants within each age-sex stratum as having low (< 25th percentile), intermediate (25th–75th percentile), or high (> 75th percentile) strength. To assess potential non-linear dose-response relationships, we conducted supplementary analyses using restricted cubic splines with 4 knots (placed at the 5th, 35th, 65th, and 95th percentiles) following Harrell’s recommendations [31]. Non-linearity was formally tested using likelihood ratio tests comparing models with and without spline terms. To examine dose-response patterns in joint exposure contexts, we modeled one exposure continuously (with splines) while stratifying by categories of the other exposure, testing for interaction using likelihood ratio tests. Participants with missing data on the exposure variables (waist circumference, grip strength), on key design variables (age group, sex, or study region) or on the outcome (incident T2D) were excluded from the analytical cohort. Missing values in covariates (education, socioeconomic status, smoking, alcohol, healthy diet and sedentary behavior) were handled using missing indicator categories in the primary analyses. To assess the influence of missing values, we conducted multiple imputation using chained equations (10 datasets with 5 iterations each) [36].

All data processing and statistical analyses were performed using R V.4.5.1 [37]. For Cox regression, we used the rms package [38]. All additive interaction measures were computed using the msm package [39]. All p-values were two-sided, and values < 0.05 were considered statistically significant.

## Results

Our analytical cohort included individuals with a mean age of 56.4 ± 8.1 years at baseline, of whom 55% were women. Participants with a low waist circumference and higher grip strength were younger, had higher educational attainment and socioeconomic status, were less likely to smoke, consumed a higher-quality diet, were more likely to drink alcohol and had less sedentary time. These patterns were more pronounced among women. Educational and socioeconomic gradients were also steeper in women: the proportion with a college degree declined from 43% among those with low waist circumference and high grip strength to 22% among those with high waist circumference and low grip strength, compared with a smaller decline in men (41% to 26%). By comparison, men showed higher absolute levels of smoking, alcohol use, and poor diet quality, whereas women reported less sedentary time (Table 1a and 1b).

**Table 1:** a. Baseline characteristics by the combination of waist circumference and grip strength in men. b. Baseline characteristics by the combination of waist circumference and grip strength in women

	Waist circumference								
	Low			Intermediate			High		
Grip strength	High	Intermediate	Low	High	Intermediate	Low	High	Intermediate	Low
**a**									
**N**	31,122	33,924	33,730	21,789	20,492	19,443	19,796	18,293	19,538
**Age (years, SD)**	55.7 (8.33)	55.6 (8.37)	55.6 (8.38)	56.8 (8.07)	57.1 (8.04)	57.2 (8.08)	57.2 (7.78)	57.6 (7.77)	57.7 (7.80)
**GS (kg, SD)**	51.7 (5.4)	42.7 (3.3)	33.4 (5.3)	51.8 (5.7)	42.3 (3.3)	32.9 (5.4)	51.9 (5.9)	42.1 (3.2)	32.3 (5.7)
**WC (cm, SD)**	87.7 (5.1)	87.2 (5.4)	86.7 (5.7)	98.3 (2.3)	98.2 (2.3)	98.2 (2.3)	110.4 (7.5)	110.6 (7.8)	111.3 (8.2)
**Education level (%)**									
College	40.8	40.6	39.6	33.6	33.2	32.4	28.1	26.9	26.4
A Level	10.9	11.0	10.6	10.8	10.8	10.1	10.6	10.3	10.3
O Level	18.3	18.4	17.1	19.9	19.6	18.6	21.1	20.9	18.3
CSE	6.2	6.0	6.2	6.6	6.5	6.1	6.8	6.7	6.6
NVQ	8.7	7.8	6.9	10.0	9.2	7.9	10.3	9.3	8.6
Other	3.6	3.5	3.3	4.5	4.1	4.0	4.6	4.7	4.2
None	10.2	11.2	13.9	13.0	14.8	18.3	16.7	19.0	22.6
Missing	1.3	1.6	2.3	1.6	1.8	2.5	1.8	2.1	2.9
**Socioeconomic status (%)**									
Q1	22.9	20.6	16.9	23.0	20.8	16.9	20.5	18.3	14.7
Q2	22.0	20.3	16.9	21.8	21.0	17.7	20.6	18.7	15.6
Q3	20.2	19.5	18.3	21.0	19.7	18.8	19.9	20.0	17.8
Q4	18.3	19.8	21.3	19.0	19.1	20.3	19.9	20.2	22.1
Q5	16.4	19.7	26.4	15.2	19.2	26.2	18.9	22.7	29.5
Missing	0.1	0.2	0.1	0.1	0.2	0.1	0.1	0.1	0.2
**Smoking status (%)**									
Never	56.1	55.9	55.9	48.8	49.1	49.5	42.9	43.5	45.3
Previous	31.3	30.1	28.1	39.2	38.0	36.2	44.4	43.3	41.1
Current	12.3	13.6	15.5	11.6	12.5	13.6	12.1	12.5	12.8
Missing	0.3	0.3	0.6	0.3	0.4	0.7	0.5	0.6	0.8
**Alcohol use (%)**									
Never	1.9	2.5	4.3	1.6	2.3	4.1	2.0	2.4	4.0
Previous	2.3	3.0	4.1	2.3	2.7	4.3	3.1	3.5	5.1
Current	95.7	94.4	91.1	96.0	94.8	91.1	94.7	93.9	90.4
Missing	0.1	0.2	0.5	0.1	0.2	0.5	0.2	0.2	0.5
**Diet quality (%)**									
Low	26.8	28.4	31.7	31.1	32.1	34.6	34.8	36.5	38.5
Moderate	66.6	65.3	62.3	65.0	64.2	61.6	62.2	60.8	58.8
High	6.5	6.3	5.7	3.8	3.6	3.6	2.9	2.6	2.4
Missing	0.1	0.1	0.3	0.1	0.1	0.2	0.1	0.1	0.3
**Sedentary time (%)**									
<4 h/d	37.2	37.4	38.1	26.4	27.0	27.8	19.3	20.1	21.7
4–<6 h/d	34.2	33.7	32.3	35.8	35.4	34.3	33.7	32.3	31.1
6–<8 h/d	17.1	17.4	16.9	21.5	21.3	20.9	25.2	25.0	23.9
≥8 h/d	11.4	11.3	12.3	16.2	16.1	16.7	21.7	22.4	22.8
Missing	0.1	0.2	0.4	0.1	0.1	0.3	0.2	0.2	0.5
**b**									
**N**	37,215	39,165	36,136	22,229	21,979	21,487	29,038	27,340	30,862
**Age (years, SD)**	54.9 (8.0)	55.1 (8.1)	55.1 (8.1)	56.8 (7.9)	56.9 (7.8)	57.0 (7.9)	57.2 (7.7)	57.3 (7.7)	57.2 (7.7)
**GS (kg, SD)**	32.3 (3.9)	26.0 (2.7)	19.5 (4.0)	31.9 (4.1)	25.5 (2.8)	18.9 (4.0)	31.9 (4.2)	25.3 (2.7)	18.4 (4.2)
**WC (cm, SD)**	73.6 (4.6)	73.3 (4.7)	73.3 (4.9)	84.3 (2.3)	84.3 (2.3)	84.4 (2.3)	98.6 (8.8)	98.2 (8.4)	99.0 (9.0)
**Education level (%)**									
College	43.3	38.3	33.4	35.2	30.6	26.9	30.4	26.0	21.6
A Level	13.7	13.3	12.7	13.0	11.9	11.2	12.2	10.9	10.1
O Level	21.2	23.0	23.7	23.2	23.9	23.7	23.1	24.3	23.3
CSE	4.3	5.3	6.6	5.1	6.0	6.9	5.7	6.5	7.5
NVQ	3.2	3.6	3.9	4.0	4.6	4.9	4.7	5.3	5.8
Other	4.9	4.7	4.8	5.7	5.5	5.2	6.3	5.8	5.5
None	8.2	10.3	13.2	12.2	15.6	19.1	16.0	19.1	23.3
Missing	1.3	1.5	1.8	1.6	2.0	2.1	1.8	2.1	2.8
**Socioeconomic status (%)**									
Q1	23.2	21.9	19.8	21.3	20.1	17.5	18.2	16.5	14.3
Q2	21.5	20.6	19.7	21.1	20.1	19.3	18.8	18.4	15.9
Q3	20.5	20.7	20.1	21.1	20.7	20.0	19.7	19.5	18.2
Q4	19.1	19.8	20.7	19.6	20.4	21.0	20.9	21.8	21.9
Q5	15.6	16.8	19.6	16.8	18.6	22.0	22.3	23.6	29.6
Missing	0.1	0.1	0.2	0.1	0.1	0.1	0.1	0.2	0.1
**Smoking status (%)**									
Never	62.1	63.1	63.3	57.4	58.9	61.2	55.4	56.6	58.2
Previous	28.8	27.8	26.3	32.8	31.4	28.7	34.6	33.3	31.2
Current	8.8	8.8	10.0	9.4	9.3	9.4	9.6	9.6	9.7
Missing	0.3	0.3	0.4	0.3	0.5	0.6	0.4	0.6	0.9
**Alcohol use (%)**									
Never	3.5	4.3	6.0	3.9	4.7	7.4	5.3	6.3	9.7
Previous	2.4	2.8	3.7	2.5	2.8	3.8	3.8	4.0	5.3
Current	94.0	92.7	90.1	93.5	92.3	88.4	90.7	89.5	84.5
Missing	0.1	0.1	0.3	0.1	0.2	0.5	0.2	0.2	0.5
**Diet quality (%)**									
Low	11.7	13.3	15.2	13.7	15.0	17.2	17.3	18.4	20.7
Moderate	80.2	79.3	77.9	80.4	80.0	77.9	78.1	77.5	75.2
High	8.0	7.3	6.8	5.8	4.9	4.7	4.5	4.0	3.9
Missing	0.1	0.1	0.1	0.1	0.1	0.3	0.1	0.1	0.3
**Sedentary time (%)**									
<4 h/d	55.5	53.0	50.2	44.9	43.0	41.0	35.8	34.5	33.2
4–<6 h/d	30.4	31.7	32.4	35.5	35.2	35.8	36.4	36.7	35.6
6–<8 h/d	9.8	10.4	11.6	13.6	14.8	15.1	18.3	18.3	18.9
≥8 h/d	4.2	4.8	5.6	6.0	6.8	7.6	9.4	10.3	11.7
Missing	0.1	0.1	0.2	0.1	0.2	0.5	0.2	0.2	0.6

Values are age-standardized mean and SDs for continuous variables (except for age and N) or percentages for categorical variables. Age standardization was done by direct standardization to the age distribution of the cohort at baseline

GS (kg) was categorized into age- and sex-specific tertiles (Additional file 1: Table S1). WC was categorized into sex-specific tertiles according to World Health Organization thresholds: WC high: >88 cm for women; WC intermediate: 81–88 cm; WC low: ≤80 cm. The socioeconomic status was calculated by the Townsend deprivation index which is constructed from the following four variables: households without a car, overcrowded households, households not owner-occupied, persons unemployed. The diet quality was calculated by the Healthy Eating Index, a scoring metric that can be used to determine overall diet quality as well as the quality of several dietary components (Additional file 1: Table S2)

*A Level* Advanced Levels/Advanced Subsidiary Levels or equivalent, *College* College or University degree, *CSE* Certificate of Secondary Education or equivalent, *GS* Grip Strength, *None* None of the above, *NVQ* National Vocational Qualification or Higher National Diploma or Higher National Certificate or equivalent, *O Level* Ordinary Levels/General Certificate of Education or equivalent, *Other* Other professional qualifications e.g., nursing, teaching, *Q1* Least deprived, *Q2* Less deprived, *Q3* Moderately deprived, *Q4* More deprived, *Q5* Most deprived, *SD* Standard Deviation, *WC* Waist Circumference

Over a follow-up of 13.0 years (totaling 6,278,500 person-years), 30,240 participants (6.3%) developed T2D. Formal tests of the proportional hazards assumptions indicated statistically detectable, but modest, departures from proportionality for the main exposure and several covariates (all global *p* < 0.001; Additional file 1: Table S3, Figure S3, Figure S4).

### Relationship of waist circumference and grip strength with T2D

**Fig. 1:**
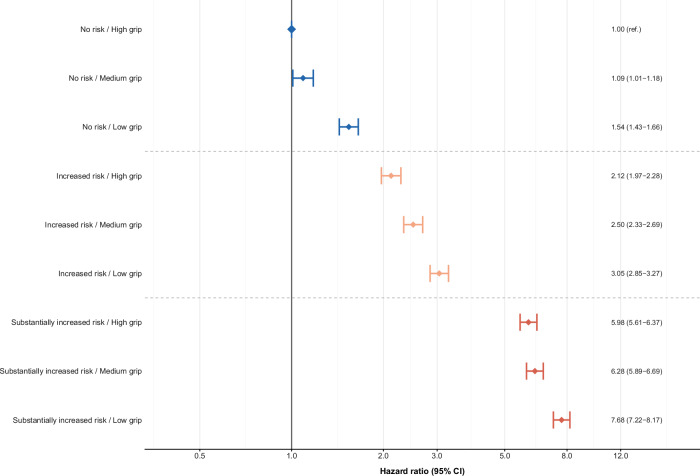
Joint associations of waist circumference and grip strength in relation to type 2 diabetes. Cox proportional hazards models used age as the underlying time scale. Models were stratified by study region, sex and age group, and adjusted for education, socioeconomic status, smoking, alcohol, healthy diet score and sedentary behavior. Waist circumference cut-offs for men: Substantially increased risk: > 102 cm; Increased risk: 95–102 cm; No risk: ≤ 94 cm. Waist circumference cut-offs for women: Substantially increased risk: > 88 cm; Increased risk: 81–88 cm; No risk: ≤ 80 cm. Reference category: No risk (waist circumference)/ High grip strength (HR = 1.00)

In mutually adjusted separate analyses, participants with high waist circumference had an HR of 5.48 (95% CI 5.30–5.66) compared with those with low waist circumference, and participants with low grip strength had an HR of 1.35 (95% CI 1.32–1.39) compared with those with high grip strength (Additional file 1: Table S4). In joint analyses, compared with individuals with low waist circumference and high grip strength, participants with high waist circumference and low grip strength had the highest risk of developing T2D (HR 7.68; 95% CI 7.22–8.17; *p* for interaction < 0.0001) (Fig. 1, Additional file 1: Table S4). The interaction on the multiplicative scale indicated a sub-multiplicative pattern, with the relative increase in T2D risk associated with low grip strength smaller among participants with high waist circumference (28% higher risk; HR 7.68 vs. 5.98) than among those with low waist circumference (54% higher risk; HR 1.54 vs. 1.00). On the additive scale, the combination of high waist circumference and low grip strength produced a RERI of 0.65 beyond additivity, with 9% of the total excess risk attributable to the joint effect itself and a small degree of synergy (Additional file 1: Table S5). In supplementary spline analyses, waist circumference showed a non-linear association with T2D risk, whereas grip strength was largely linear, with significant interaction observed in joint models (Additional file 1: Figure S5, Figure S6).

### Sex-specific relations of waist circumference and grip strength to T2D

In sex-stratified analyses, high versus low waist circumference was associated with HRs of 4.48 (95% CI 4.30–4.66) in men and 7.72 (95% CI 7.28–8.19) in women (*p* for interaction < 0.0001), and low versus high grip strength was associated with HRs of 1.42 (95% CI 1.37–1.47) in men and 1.25 (95% CI 1.20–1.31) in women (*p* for interaction = 0.0018) (Table 2). When considering the joint phenotype, the association of high waist circumference and low grip strength with T2D was stronger in women (HR 10.24; 95% CI 9.16–11.46) than in men (HR 6.47; 95% CI 6.00–6.98; *p* for interaction < 0.0001), consistent with sex differences in the individual waist circumference and grip strength associations with T2D. The three-way interaction (waist circumference × grip strength × sex) (*p* = 0.6000) indicated that the sub-multiplicative interaction observed in the overall cohort was similar in men and women. On the additive scale, the joint association of high waist circumference and low grip strength exceeded the sum of their individual associations in both sexes, with 17% of the excess risk in men and 13% in women attributable to interaction (M: RERI 1.09; AP 0.17; S 1.25; W: RERI 1.32; AP 0.13; S 1.17) (Additional file 1: Table S6).

**Table 2:** Waist circumference, grip strength, and the combination of waist circumference and grip strength in relation to T2D, stratified by sex

	Men				Women			
	Cases	PYs	HR	95% CI	Cases	PYs	HR	95% CI
**WC**								
Low	3353	1,293,949	1.00	–	1299	1,508,699	1.00	–
Intermediate	4479	792,232	1.99	1.90–2.08	2055	872,087	2.39	2.23–2.56
High	9683	699,847	4.48	4.30–4.66	9371	1,111,685	7.72	7.28–8.19
	*p* for interaction (WC x sex) < 0.0001							
**GS**								
High	4900	946,640	1.00	–	3625	1,180,217	1.00	–
Intermediate	5388	933,860	1.11	1.07–1.15	3818	1,167,492	1.04	1.00–1.09
Low	7227	905,527	1.42	1.37–1.47	5282	1,144,763	1.25	1.20–1.31
	*p* for interaction (GS x sex) = 0.0018							
**Low WC**								
High GS	829	414,720	1.00	–	341	505,272	1.00	–
Intermediate GS	1029	446,651	1.11	1.01–1.22	397	525,223	1.04	0.90–1.20
Low GS	1495	432,578	1.55	1.42–1.69	561	478,204	1.49	1.31–1.71
**Intermediate WC**								
High GS	1226	285,111	1.95	1.79–2.13	578	298,721	2.49	2.18–2.85
Intermediate GS	1459	263,300	2.40	2.20–2.61	658	292,012	2.72	2.39–3.10
Low GS	1794	243,821	2.96	2.73–3.22	819	281,354	3.25	2.86–3.69
**High WC**								
High GS	2845	246,809	4.83	4.47–5.22	2706	376,223	8.42	7.52–9.43
Intermediate GS	2900	223,910	5.13	4.74–5.54	2763	350,257	8.71	7.77–9.75
Low GS	3938	229,128	6.47	6.00–6.98	3902	385,205	10.24	9.16–11.46
	*p* for interaction (combination of WC and GS x sex) < 0.0001							

Models used age as the underlying time scale. Models were stratified by study region and age group, and adjusted for education, socioeconomic status, smoking, alcohol, healthy diet score and sedentary behavior. For analyses of separate relations, waist circumference and grip strength were mutually adjusted. *p*-value for interaction derived from a Likelihood-Ratio test

*WC* Waist Circumference, *GS* Grip Strength, *T2D* Type 2 diabetes, *PYs* Person-Years, *HR* Hazard Ratio, *CI* Confidence Interval

### Age-specific relations of waist circumference and grip strength to T2D

In age-stratified analyses, high versus low waist circumference was more strongly associated with T2D in adults younger than 60 years (HR 6.72; 95% CI 6.39–7.06) than in those aged 60 years or older (HR 4.51; 95% CI 4.32–4.71; *p* for interaction < 0.0001). Low versus high grip strength showed HRs of 1.43 (95% CI 1.37–1.49) in younger adults and 1.30 (95% CI 1.25–1.35) in older adults (*p* for interaction < 0.0001) (Table 3). When waist circumference and grip strength were evaluated jointly, the association of high waist circumference and low grip strength with T2D was stronger in participants younger than 60 years (HR 10.12; 95% CI 9.17–11.17) than in those aged 60 years or older (HR 6.04; 95% CI 5.57–6.54; *p* for interaction < 0.0001), and this difference corresponds to the age-related variation in the individual waist and grip associations. The three-way interaction (waist circumference × grip strength × age) (*p* = 0.69), indicated that the sub-multiplicative pattern seen overall was comparable across age strata. On the additive scale, a larger proportion of the excess risk was attributable to interaction in younger adults (19%) than in older adults (11%) (younger: RERI 1.95; AP 0.19; S 1.27; older: RERI 0.65; AP 0.11; S 1.15) (Additional file 1: Table S7).

**Table 3:** Waist circumference, grip strength, and the combination of waist circumference and grip strength in relation to T2D, stratified by age

Age	< 60 Years				≥ 60 Years			
	Cases	PYs	HR	95% CI	Cases	PYs	HR	95% CI
**WC**								
Low	1967	1,781,880	1.00	–	2685	1,020,768	1.00	–
Intermediate	2553	927,424	2.21	2.08–2.34	3981	736,895	1.97	1.87–2.07
High	8705	985,536	6.72	6.39–7.06	10,349	825,996	4.51	4.32–4.71
	*p* for interaction (WC x age) < 0.0001							
**GS**								
High	3570	1,249,153	1.00	–	4955	877,703	1.00	–
Intermediate	3889	1,235,454	1.11	1.06–1.16	5317	865,899	1.07	1.03–1.11
Low	5766	1,210,233	1.43	1.37–1.49	6743	840,057	1.30	1.25–1.35
	*p* for interaction (GS x age) < 0.0001							
**Low WC**								
High GS	455	581,730	1.00	–	715	338,262	1.00	–
Intermediate GS	593	617,769	1.14	1.01–1.29	833	354,105	1.06	0.96–1.18
Low GS	919	582,381	1.67	1.49–1.87	1137	328,401	1.48	1.34–1.62
**Intermediate WC**								
High GS	675	326,686	2.26	2.01–2.55	1129	257,146	1.96	1.79–2.16
Intermediate GS	786	309,221	2.65	2.36–2.98	1331	246,091	2.33	2.13–2.55
Low GS	1092	291,517	3.53	3.16–3.94	1521	233,658	2.68	2.45–2.93
**High WC**								
High GS	2440	340,737	7.50	6.79–8.30	3111	282,295	4.91	4.52–5.33
Intermediate GS	2510	308,463	8.12	7.34–8.98	3153	265,704	5.06	4.66–5.49
Low GS	3755	336,335	10.12	9.17–11.17	4085	277,998	6.04	5.57–6.54
	*p* for interaction (combination of WC and GS x age) < 0.0001							

Models used age as the underlying time scale. Models were stratified by study region and sex, and adjusted for education, socioeconomic status, smoking, alcohol, healthy diet score, and sedentary behavior. For analyses of separate relations, waist circumference and grip strength were mutually adjusted. *p*-value for interaction derived from a Likelihood-Ratio test

*WC* Waist Circumference, *GS* Grip Strength, *T2D* Type 2 diabetes, *PYs* Person-Years, *HR* Hazard Ratio, *CI* Confidence Interval

### Additional analyses

After additional adjustment for physical activity, height, and family history of diabetes, associations were slightly attenuated but remained materially unchanged (Additional file 1: Table S8). Compared with the reference group, individuals with the combination of high waist circumference and low grip strength had an almost sevenfold higher hazard of developing T2D (HR: 6.86; 95% CI 6.44–7.30). Applying alternative age- and sex-specific cut-offs for normalized grip strength yielded similar patterns, with HRs ranging from 5.69 to 6.70 in the high waist circumference group (Additional file 1: Table S9). Using Tomkinson cut-offs for absolute grip strength produced comparable results (Additional file 1: Table S10). Results from the multiple imputation analyses were virtually identical to those obtained from the missing-indicator and complete-case analyses, with no material differences in HRs or CIs (Additional file 1: Table S11).

## Discussion

To the best of our knowledge, this is the first prospective study in a large European cohort to jointly examine abdominal obesity and handgrip strength in relation to incident diabetes. Higher waist circumference and lower handgrip strength were each separately associated with increased diabetes risk. While these individual associations are well documented, the novelty of our study lies in their joint evaluation and in the clinical interpretation of their combined effects. By demonstrating excess risk on the additive scale among individuals with both high waist circumference and low handgrip strength, our findings identify a distinct high-risk phenotype characterized by the coexistence of elevated metabolic load and reduced metabolic reserve, providing a basis for more refined risk stratification beyond either factor considered in isolation, though the clinical added value of joint assessment warrants evaluation in formal prediction studies. Abdominal obesity conferred substantial risk even among individuals with high muscular strength, whereas low muscular strength increased risk even among abdominally lean participants. Although the association between grip strength and diabetes was successively attenuated at increasing levels of waist circumference, indicating a sub-multiplicative interaction, additive-scale estimates suggested excess risk among individuals jointly exhibiting high waist circumference and low muscular strength. By explicitly evaluating interaction on both multiplicative and additive scales, our analyses address a methodological gap in the existing literature, which has largely focused on independent associations or relative risks without quantifying excess risk attributable to joint exposure.

Research on the combined role of abdominal adiposity and handgrip strength in diabetes risk is limited to one Asian prospective study [18] and one multicountry cross-sectional study [40]. Both identified abdominal obesity as the key factor associated with diabetes, with similar risk estimates for abdominal obesity alone and for the combined phenotype. Findings from two additional studies on related metabolic outcomes, a European prospective study on metabolic syndrome [41] and a South American cross-sectional study on glycated hemoglobin (HbA1c) [42], mirror the pattern we observed for diabetes risk by showing that excess abdominal adiposity combined with low handgrip strength was associated with more adverse metabolic characteristics than either factor alone.

A related line of work examined general obesity in combination with handgrip strength in relation to diabetes. One European prospective study found higher diabetes risk among individuals with both general obesity and low handgrip strength compared with either factor alone [32]. Adding a related nuance, two Asian cross-sectional studies reported that inverse associations of handgrip strength with prediabetes [43] and diabetes [44] were confined to individuals without general obesity, suggesting that in leaner individuals, variation in handgrip strength may reflect metabolic vulnerability otherwise masked by excess adiposity.

Biological, interventional, and genetic evidence supports these findings. Visceral adiposity promotes insulin resistance through dysregulated adipokines, elevated free fatty acid flux, and chronic inflammation [7]. Grip strength likely reflects underlying muscle quantity, quality, or cardiorespiratory fitness rather than grip strength per se [15], and these attributes are closely linked to mitochondrial efficiency, muscle fiber composition, neuromuscular function, and intramuscular fat infiltration [45]. Experimental studies show that improving muscle strength enhances these underlying attributes and improves insulin sensitivity independent of weight loss [46]. Systematic reviews of randomized trials further confirm that resistance and combined aerobic-resistance exercise lower HbA1c [47]. Mendelian randomization suggests a potentially causal role for muscular strength in diabetes risk [48]. Together, these data reinforce the separate and joint roles of abdominal adiposity and muscular fitness in metabolic regulation.

We found that the positive waist circumference-diabetes association was more pronounced in women than men, consistent with prospective evidence from Europe [49] and Asia [50]. In contrast, we observed a more pronounced inverse handgrip strength-diabetes relation in men than women. While prior European prospective data suggest little sex difference in the strength-diabetes relation [14], our findings align with Asian longitudinal evidence suggesting a stronger inverse association of grip strength to diabetes risk in men than women [51, 52].

These sex-specific patterns are biologically plausible. Increments in visceral fat area confer disproportionately higher T2D risk in women than in men [53]. This greater susceptibility likely reflects sex hormone-dependent differences in adipose function [54], including changes emerging during the menopausal transition [55]. Conversely, men have greater absolute lean mass and muscular strength than women [56], and greater muscle mass is associated with less insulin resistance, even after adjusting for adiposity [57].

We noted that the waist circumference-diabetes association was markedly stronger in adults younger than 60 years than in those aged 60 years and older. This pattern agrees with European cohort data, where the discriminatory performance of waist-based diabetes risk scores was higher before age 60 [58], and with Asian prospective evidence showing steeper adiposity-diabetes gradients in midlife than in later adulthood, although those analyses used body mass index (BMI) rather than waist circumference [59]. Our data also showed that grip strength demonstrated a stronger inverse association with diabetes in younger than older adults, a pattern also observed in longitudinal studies from Europe [14] and Asia [51, 52].

Several mechanisms may explain the steeper adiposity-diabetes gradient in younger adults. Earlier onset and longer duration of obesity impose prolonged metabolic stress [60], whereas ageing brings declining β-cell function [61] and redistribution of fat toward visceral depots [62], such that hyperglycemia in older adults may reflect primary β-cell failure rather than incremental increases in abdominal adiposity. Age-related changes in muscle mass and quality provide a parallel explanation for grip strength. In younger adults, greater muscle mass and better muscle quality are strongly related to insulin sensitivity, so lower strength may more directly signal impaired glucose disposal capacity [63]. With ageing, generalized sarcopenia, intramuscular fat infiltration, comorbidity, and inactivity become more prevalent [64] and may dominate variation in strength, weakening the link between grip strength and diabetes.

Our findings have important implications for public health and clinical practice. Routine joint assessment of waist circumference and handgrip strength is appealing given that both measures are low-cost and scalable, however, formal evaluation of their incremental predictive utility beyond established risk factors is needed before recommending their combined use for clinical risk stratification. While abdominal adiposity plays a central role in diabetes prevention, grip strength adds meaningful and complementary information for risk stratification by identifying elevated risk even among individuals without abdominal obesity and should be interpreted primarily as a proxy for overall muscular fitness and lean mass rather than a direct causal determinant of T2D. The more-than-additive joint association observed in our study raises the possibility that among individuals with excess abdominal adiposity and low muscular strength, jointly addressing both factors may confer greater benefit than targeting either alone, although this hypothesis requires confirmation in intervention studies and should be interpreted cautiously given the modest magnitude of the additive excess risk. Our sex-specific findings further indicate that greater emphasis on body fat reduction in women and strength enhancement in men may refine risk assessment, although both targets remain clinically relevant in both sexes, and the stronger associations observed in younger adults underscore the importance of prevention efforts beginning in early adulthood and continuing through midlife. Because resistance training can improve strength and glycemic control even without reducing adiposity, preventive strategies should aim to target both metabolic load through adiposity reduction and metabolic reserve through strength enhancement concurrently. Collectively, our findings support the concept of dynapenic abdominal obesity - the coexistence of reduced muscular strength and abdominal adiposity - as a clinically relevant phenotype associated with elevated risks of T2D.

### Strengths and limitations

Strengths of our study are its prospective design, large sample size, standardized anthropometric and muscular strength assessments, comprehensive adjustment for confounding variables, and registry-based ascertainment of T2D, which ensured high completeness and accuracy of case identification. Importantly, by jointly examining waist circumference and grip strength, we were able to disentangle their separate and combined contributions to T2D risk and identify subgroup-specific associations by age and sex. Examining these two low-cost, scalable measures in combination represents a further strength, as waist circumference captures abdominal adiposity more precisely than BMI, and grip strength provides a practical proxy for overall muscular fitness and lean mass.

Nevertheless, several limitations should be acknowledged. First, grip strength and waist circumference were measured only once at baseline, which precluded assessment of temporal changes or training-induced improvements during follow-up. Second, residual confounding from unmeasured lifestyle or genetic factors cannot be fully excluded. Adjustment for family history of diabetes was used as a pragmatic attempt to partially account for shared genetic susceptibility, while recognizing that this approach cannot fully capture individual genetic risk. Third, formal tests of the proportional hazards assumption indicated statistical departures (Additional file 1: Table S3, Figure S3, Figure S4). Such patterns are commonly observed in large prospective cohorts, as individuals with highest metabolic risk develop diabetes or experience competing events earlier in follow-up, progressively depleting the at-risk population of the most susceptible individuals [65, 66]. Additionally, statistical tests become highly sensitive in samples of this magnitude (*n* = 483,578), detecting even minor, clinically negligible time-variation as statistically significant [67]. Our HRs should therefore be interpreted as weighted temporal averages across the follow-up period. Importantly, the direction and magnitude of associations remained robust across multiple sensitivity analyses and subgroups, supporting the validity of our findings. Fourth, our observational design precludes causal inference. Finally, generalizability is limited by the volunteer composition of the UK Biobank cohort, which is healthier and less socioeconomically diverse than the general population.

## Conclusions

In this large prospective cohort, higher waist circumference and lower grip strength were each separately associated with higher T2D risk, with their combination identifying individuals at particularly elevated risk. Their joint relation exceeded what would be expected from their individual contributions alone, and associations for waist circumference were stronger in women, whereas grip strength showed more pronounced associations in men, and both patterns more marked in younger adults. These findings suggest that the combined use of waist circumference and grip strength may contribute to diabetes risk stratification and highlight dynapenic abdominal obesity as a clinically relevant phenotype, though formal evaluation of their incremental predictive utility is warranted. Integrating these two low-cost, scalable measures into routine assessment holds promise for early identification of vulnerable individuals and may help guide preventive strategies targeting reductions in abdominal adiposity and improvements in muscular fitness, pending confirmation of their incremental predictive value. Future research should examine the causal pathways underlying these associations and evaluate the impact of targeted interventions on long-term diabetes risk.

## Supplementary information

Additional file 1 Table S1 Age- and sex-specific cut-off values (kg) for absolute GS tertiles. Table S2 Covariate details. Table S3 Global Schoenfeld residual tests of the proportional hazards assumption across Cox regression models. Table S4 WC, GS, and the combination of WC and GS in relation to T2D. Table S5 Full additive interaction measures between WC and GS in relation to T2D risk. Table S6 Additive interaction between WC and GS in relation to T2D risk, stratified by sex. Table S7 Additive interaction between WC and GS in relation to T2D risk, stratified by age. Table S8 Fully adjusted HRs and 95% CIs for the combination of WC and GS in relation to T2D. Table S9 HRs and 95% CIs for the combination of WC and NGS in relation to T2D. Table S10 HRs and 95% CIs for the combination of WC and GS (Tomkinson cut-offs) in relation to T2D. Table S11 Comparison of HRs for incident T2D across different approaches to handling missing covariate data. Figure S1 Flow chart of participant inclusion and exclusion. Figure S2 Directed acyclic graph. Figure S3 Scaled Schoenfeld residual plots for separate WC and GS models. Figure S4 Scaled Schoenfeld residual plots for the joint exposure model. Figure S5 Non-linear association between WC and T2D risk stratified by GS. Figure S6 Association between GS and T2D risk stratified by WC

Additional file 2 STROBE Statement

## Data Availability

UK Biobank is an open access resource. Bona fide researchers can apply to use UK Biobank dataset by registering and applying at http://ukbiobank.ac.uk/register-apply/.

## Abbreviations

AP: Attributable proportion
BMI: Body mass index
CI: Confidence interval
DAG: Directed acyclic graph
HbA1c: glycated hemoglobin
HR: Hazard ratio
M: Men
RERI: Relative excess risk due to interaction
S: Synergy index
T2D: Type 2 diabetes
W: Women
