# Supplementary material for: Waist circumference and grip strength and their joint relations to type 2 diabetes incidence in UK Biobank
Source: BMC Med. 2026 May 7;24:299. doi: 10.1186/s12916-026-04907-8 (PMC13154465; doi:10.1186/s12916-026-04907-8)
Supplement: Supplementary file 1 — Additional file 1 Table S1 Age- and sex-specific cut-off values (kg) for absolute GS tertiles. Table S2 Covariate details. Table S3 Global Schoenfeld residual tests of the proportional hazards assumption across Cox regression models. Table S4 WC, GS, and the combination of WC and GS in relation to T2D. Table S5 Full additive interaction measures between WC and GS in relation to T2D risk. Table S6 Additive interaction between WC and GS in relation to T2D risk, stratified by sex. Table S7 Additive interaction between WC and GS in relation to T2D risk, stratified by age. Table S8 Fully adjusted HRs and 95% CIs for the combination of WC and GS in relation to T2D. Table S9 HRs and 95% CIs for the combination of WC and NGS in relation to T2D. Table S10 HRs and 95% CIs for the combination of WC and GS (Tomkinson cut-offs) in relation to T2D. Table S11 Comparison of HRs for incident T2D across different approaches to handling missing covariate data. Figure S1 Flow chart of participant inclusion and exclusion. Figure S2 Directed acyclic graph. Figure S3 Scaled Schoenfeld residual plots for separate WC and GS models. Figure S4 Scaled Schoenfeld residual plots for the joint exposure model. Figure S5 Non-linear association between WC and T2D risk stratified by GS. Figure S6 Association between GS and T2D risk stratified by WC [file 12916_2026_4907_MOESM1_ESM.docx]

**Additional file 1: Waist circumference and grip strength and their joint relations to type 2 diabetes incidence in UK Biobank**

Table S1. Age- and sex-specific cut-off values (kg) for absolute GS tertiles

Table S2. Covariate details

Table S3. Global Schoenfeld residual tests of the proportional hazards assumption across cox regression models

Table S4. WC, GS, and the combination of WC and GS in relation to T2D

Table S5. Full additive interaction measures between WC and GS in relation to T2D risk

Table S6. Additive interaction between WC and GS in relation to T2D risk, stratified by sex

Table S7. Additive interaction between WC and GS in relation to T2D risk, stratified by age

Table S8. Fully adjusted HRs and 95% CIs for the combination of WC and GS in relation to T2D

Table S9. HRs and 95% CIs for the combination of WC and NGS in relation to T2D

Table S10. HRs and 95% CIs for the combination of WC and GS (Tomkinson cut-offs) in relation to T2D

Table S11. Comparison of HRs for incident T2D across different approaches to handling missing covariate data

Figure S1. Flow chart of participant inclusion and exclusion

Figure S2. Directed acyclic graph

Figure S3. Scaled Schoenfeld residual plots for separate WC and GS models

Figure S4. Scaled Schoenfeld residual plots for the joint exposure model

Figure S5. Non-linear association between WC and T2D risk stratified by GS

Figure S6. Association between GS and T2D risk stratified by WC

**Corresponding author:** Johanna Wirler, Tel.: +49 941 944 52 55,

Email: Johanna.wirler@ur.de, Department of Epidemiology and Preventive Medicine, University of Regensburg, 93053 Regensburg, Germany

| **Table S1. Age- and sex-specific cut-off values (kg) for absolute GS tertiles** | | | | |
| --- | --- | --- | --- | --- |
| **Age group (years)** | **Women** | | **Men** | |
|  | **Cut-off 1** | **Cut-off 2** | **Cut-off 1** | **Cut-off 2** |
| 40–44 | 26 | 32 | 42 | 50 |
| 45–49 | 25 | 30 | 40 | 48 |
| 50–54 | 24 | 29 | 40 | 48 |
| 55–59 | 22 | 28 | 39 | 46 |
| 60–64 | 21 | 26 | 37 | 44 |
| 65–69 | 20 | 25 | 35 | 42 |
| GS: Grip strength | | | | |

| **Table S2. Covariate details** | | | |
| --- | --- | --- | --- |
| **Covariate** | **UK Biobank – variable identification number** | **Calculation** | **Further Comments** |
| Age group | ID 21022: Age at recruitment | Categorized to groups with 5-year steps:  40-44, 45-49, 50-54, 55-59, 60-64, 65-69 | “Prefer not to answer” were coded NA |
| Alcohol use status | ID 20117: Alcohol drinker status | Self-reported alcohol drinker status: never, previous, current |  |
| Diet | ID 1289: Cooked vegetable intake  ID 1299: Salad/raw vegetable intake  ID 1309: Fresh fruit intake  ID 1319: Dried fruit intake  ID 1329: Oily fish intake  ID 1339: Non-oily fish intake  ID 1349: Processed meat intake  ID 1359: Poultry intake  ID 1369: Beef intake  ID 1379: Lamb/Mutton intake  ID 1389: Pork intake  ID 1438: Bread intake  ID 1448: Bread type  ID 1458: Cereal intake  ID 1468: Cereal type | Building a healthy diet score based on Lourida et al. [29] ranging from 0 – 7 by giving one point per fulfilled nutritional category:   - Fruits: 3 servings/day - Vegetables: 3 servings/day - Fish: 2 servings/week - Processed meats: 1 serving/week - Unprocessed red meat: 1.5 servings/week - Whole grains: 3 servings/day - Refined grains: 1.5 servings/day | No inclusion of dairy intake (ID 1408, ID 1418, ID 1428)  Corrections:   - Vegetables & Fruit: <0 = 0; >5=5 |
| Education | ID 6138: Qualifications | Categorization in four groups:  1 = University or College degree  2 = A-level/professional qualification/HNC/NVQ  3 = 0-levels/CSE  4 = None | “Prefer not to answer” was coded NA |
| Family history of diabetes | ID 20107: Illnesses of father  ID 20110: Illnesses of mother | Family history of diabetes was yes, when any was true  9 = Diabetes |  |
| Height | ID 50: Standing height | Measured in cm |  |
| Sedentary behavior | ID 1070: Time spent watching TV  ID 1080: Time spent using computer  ID 1090: Time spent driving | Total sedentary behavior was calculated by adding up time spent watching television, time spent using computer and time spent driving.  Sedentary behavior while working was not included in our calculation. | The data was corrected by a truncation to 24h, if the sum was >24h.  Whenever there was a missing in either variable, the total sedentary behavior was considered as missing (n = 19,038) |
| Self-reported physical activity | ID 864: Number of days/week walked 10+ minutes  ID 874: Duration of walks  ID 884: Number of days/week of moderate physical activity 10+ minutes  ID 894: Duration of moderate activity  ID 904: Number of days/week of vigorous physical activity 10+ minutes  ID 914: Duration of vigorous activity | Categorization based on age- and sex-standardized tertiles of total physical activity per week based on:  Various continuous scores:   - Walking MET-min/week =   3.3 * walking minutes * walking days   - Moderate MET-min/week =   4 * moderate minutes * moderate days   - Vigorous MET-min/week = 8 * vigorous minutes * vigorous days   Total score:  MET-min/week = Walking + Moderate + Vigorous MET-min/week scores | The data were corrected by truncation of high values (>180 was set to 180 for each walking duration, vigorous and moderate physical activity).  Outliers were defined as individuals with a sum of walking, moderate and vigorous physical activity of above 960 minutes per day.  Responses below 10 minutes were set to 0.  Whenever there was a missing variable, the total physical activity was considered missing (n = 118,333)  These corrections were based on UK Biobank IPAQ Guidelines [30]. |
| Smoking status | ID 20116: Smoking status | Self-reported smoking status: never, previous, current | “Prefer not to answer” were coded NA |
| Socio-economic status | ID 22189: Townsend deprivation index at recruitment | A composite score of employment, ownership of car and home, household overcrowding and postcode; higher values indicate a higher degree of deprivation. | The calculation happens prior to participating.  The values are rounded to 2 decimal places. |
| Study region | ID 54: UK Biobank Assessment centre | The 22 single centers were grouped according to their country of origin. |  |
| CSE: Certificate of Secondary Education; HNC: Higher National Certificate; MET: Metabolic equivalent of task; NVQ: National Vocational Qualification | | | |

| **Table S3. Global Schoenfeld residual tests of the proportional hazards assumption across cox regression models** | | | | | |
| --- | --- | --- | --- | --- | --- |
| **Model** | **Description** | **Global χ²** | **df** | ***p*-value** | **Main Violations (χ², *p* < 0.001)** |
| 1 | joint exposure, stratified | 472.00 | 8 | < 0.001 | joint exposure (472.00) |
| 2 | joint exposure, stratified and adjusted | 620.25 | 33 | < 0.001 | joint exposure (451.51), socioeconomic status (138.99), alcohol (93.86) |
| 3 | joint exposure, stratified and max. adjusted | 620.30 | 45 | < 0.001 | joint exposure (406.04), socioeconomic status (133.63), physical activity (76.91) |
| 4 | WC + GS, stratified and adjusted | 605.79 | 29 | < 0.001 | WC (379.25), socioeconomic status (139.56), alcohol (94.06), GS (60.83) |
| 5 | WC + GS, stratified and max. adjusted | 606.35 | 41 | < 0.001 | WC (342.54), socioeconomic status (134.14), physical activity (77.43), GS (51.72) |
| 6 | WC * GS, stratified and adjusted | 620.25 | 33 | < 0.001 | WC (381.67), WC * GS (268.78), socioeconomic status (138.99), alcohol (93.86) |
| 7 | WC * sex, stratified and adjusted | 620.95 | 32 | < 0.001 | WC3 (359.89), WC2 (103.65), WC3 * sex (99.01), WC2 * sex (63.26) |
| 8 | GS * sex, stratified and adjusted | 606.01 | 32 | < 0.001 | WC3 (361.96), WC2 (101.43), GS3 (59.12), GS3 * sex (24.17) |
| 9 | joint exposure * sex, stratified and adjusted | 647.18 | 42 | < 0.001 | joint exposure (444.29), joint exposure * sex (220.48), socioeconomic status (136.92) |
| 10 | WC * GS * sex, stratified and adjusted | 647.18 | 42 | < 0.001 | WC3 (362.40), WC * G3 (167.02), WC3 * sex (100.94), WC3 * GS3 * sex (71.38) |
| 11 | WC + GS, stratified and adjusted (males only) | 288.07 | 29 | < 0.001 | WC3 (168.94), socioeconomic status (60.39), alcohol (46.02), WC2 (40.63) |
| 12 | WC + GS, stratified and adjusted (females only) | 363.61 | 29 | < 0.001 | WC3 (213.87), socioeconomic status (84.78), WC2 (72.51), alcohol (47.58) |
| 13 | joint exposure, stratified and adjusted (males only) | 291.74 | 33 | < 0.001 | joint exposure (208.13), socioeconomic status (60.11), alcohol (45.84) |
| 14 | joint exposure, stratified and adjusted (females only) | 384.72 | 33 | < 0.001 | joint exposure (272.58), socioeconomic status (84.68), alcohol (47.68), sedentary category (28.79) |
| 15 | WC * age, stratified and adjusted | 702.72 | 32 | < 0.001 | age (352.87), socioeconomic status (138.97), WC3 (109.54), WC2 * age (51.79) |
| 16 | GS * age, stratified and adjusted | 907.34 | 32 | < 0.001 | age (353.97), WC3 (322.31), socioeconomic status (150.60), GS2 * age (26.57) |
| 17 | joint exposure * age, stratified and adjusted | 691.39 | 42 | < 0.001 | age (352.41), joint exposure * age (186.56), joint exposure (153.88) |
| 18 | WC * GS * age, stratified and adjusted | 691.40 | 42 | < 0.001 | age (352.41), WC3 (108.78), WC3 * GS3 (57.31), WC2 * age (51.81) |
| 19 | WC + GS, stratified and adjusted (age <60 years) | 103.64 | 29 | < 0.001 | WC3 (38.39), socioeconomic status (37.02) |
| 20 | WC + GS, stratified and adjusted (age ≥60 years) | 149.74 | 29 | < 0.001 | WC3 (73.18), WC2 (26.70), G3 (25.29) |
| 21 | joint exposure, stratified and adjusted (age <60 years) | 117.75 | 33 | < 0.001 | joint exposure (62.00), socioeconomic status (36.84), |
| 22 | joint exposure, stratified and adjusted (age ≥60 years) | 158.42 | 33 | < 0.001 | joint exposure (107.22) |
| df: degrees of freedom, GS: Grip strength, GS2: second tertile of grip strength, GS3: third tertile of grip strength, WC: Waist circumference, WC2: Intermediate waist circumference (increased risk), WC3: High waist circumference (substantially increased risk) Models were stratified by study region, age and sex, and adjusted for education, socioeconomic status, smoking, alcohol, healthy diet score, and sedentary behavior. Maximally adjusted model was stratified by study region, age and sex, and adjusted for education, socioeconomic status, smoking, alcohol, healthy diet score, sedentary behavior, physical activity, height and family history of diabetes. | | | | | |

| **Table S4. WC, GS, and the combination of WC and GS in relation to T2D** | | | | | | |
| --- | --- | --- | --- | --- | --- | --- |
|  | **Cases** | **Person-years** | **HR** | **95% CI** | | |
| **WC** | | | | | | |
| Low | 4,652 | 2,802,648 | 1.00 | – | | |
| Intermediate | 6,534 | 1,664,320 | 2.11 | 2.03–2.19 | | |
| High | 19,054 | 1,811,532 | 5.48 | 5.30–5.66 | | |
| **GS** | | | | | | |
| High | 8,525 | 2,126,857 | 1.00 | – | | |
| Intermediate | 9,206 | 2,101,353 | 1.08 | 1.05–1.11 | | |
| Low | 12,509 | 2,050,290 | 1.35 | 1.32–1.39 | | |
| **Combination of WC and GS** | | | | | | |
|  | **Cases** | **Person-years** | **HR** | **95% CI** | **RERI** | **AP** |
| **Low WC** | | | | |  |  |
| High GS | 1,170 | 919,992 | 1.00 | – | – | – |
| Intermediate GS | 1,426 | 971,874 | 1.09 | 1.01–1.18 | – | – |
| Low GS | 2,056 | 910,782 | 1.54 | 1.43–1.66 | – | – |
| **Intermediate WC** | | | | |  |  |
| High GS | 1,804 | 583,833 | 2.12 | 1.97–2.28 | – | – |
| Intermediate GS | 2,117 | 555,312 | 2.50 | 2.33–2.69 | 0.24 | 0.10 |
| Low GS | 2,613 | 525,175 | 3.05 | 2.85–3.27 | 0.27 | 0.10 |
| **High WC** | | | | |  |  |
| High GS | 5,551 | 623,032 | 5.98 | 5.61–6.37 | – | – |
| Intermediate GS | 5,663 | 574,167 | 6.28 | 5.89–6.69 | 0.01 | 0.00 |
| Low GS | 7,840 | 614,333 | 7.68 | 7.22–8.17 | 0.65 | 0.09 |
| *p* for interaction (WC x GS) = 0.0001 | | | | | | |
| AP: Attributable proportion, CI: Confidence interval, GS: Grip strength, HR: Hazard ratio, RERI: Relative excess risk due to interaction, T2D: Type 2 diabetes, WC: Waist circumference  Models used age as the underlying time scale. Models were stratified by study region, sex and age group and adjusted for education, socioeconomic status, smoking, alcohol, healthy diet score, and sedentary behavior. For analyses of separate relations, WC and GS were mutually adjusted. *p*-value for interaction derived from a likelihood ratio test. Additive interaction was assessed on the additive scale using the RERI and the AP, with low WC and high GS as the reference group. CIs for RERI and AP could not be estimated due to sparse data. | | | | | | |

| **Table S5. Full additive interaction measures between WC and GS in relation to T2D risk** | | | |
| --- | --- | --- | --- |
|  | **RERI (95% CI)** | **AP (95% CI)** | **S (95% CI)** |
| High WC x Low GS | 0.65 (NA) | 0.09 (NA) | 1.12 (NA) |
| Intermediate WC x Low GS | 0.27 (NA) | 0.10 (NA) | 1.18 (NA) |
| High WC x Intermediate GS | 0.01 (NA) | 0.00 (NA) | 1.00 (NA) |
| Intermediate WC x Intermediate GS | 0.24 (NA) | 0.10 (NA) | 1.21 (NA) |
| AP: Attributable proportion, CI: Confidence interval, GS: Grip strength, RERI: Relative excess risk due to interaction, S: Synergy index, T2D: Type 2 diabetes, WC: Waist circumference  Reference group: Low WC / High GS; 95 % CI could not be estimated due to sparse data. | | | |

| **Table S6. Additive interaction between WC and GS in relation to T2D risk, stratified by sex** | | | | | | |
| --- | --- | --- | --- | --- | --- | --- |
|  | **Men** | | | **Women** | | |
|  | **RERI**  **(95% CI)** | **AP**  **(95% CI)** | **S**  **(95% CI)** | **RERI**  **(95% CI)** | **AP**  **(95% CI)** | **S**  **(95% CI)** |
| High WC x Low GS | 1.09  (0.81–1.37) | 0.17  (0.13–0.21) | 1.25  (1.17–1.33) | 1.32  (0.85–1.80) | 0.13  (0.08–0.17) | 1.17  (1.10–1.23) |
| Intermediate WC x Low GS | 0.46  (0.26–0.66) | 0.16  (0.09–0.22) | 1.31  (1.14–1.47) | 0.26  (−0.08–0.59) | 0.08  (−0.02–0.18) | 1.13  (0.94–1.32) |
| High WC x Intermediate GS | 0.19  (−0.08–0.46) | 0.04  (−0.02–0.09) | 1.05  (0.98–1.12) | 0.24  (−0.23–0.71) | 0.03  (−0.03–0.08) | 1.03  (0.97–1.10) |
| Intermediate WC x Intermediate GS | 0.34  (0.15–0.52) | 0.14  (0.07–0.22) | 1.32  (1.09–1.54) | 0.19  (−0.13–0.51) | 0.07  (−0.05–0.19) | 1.12  (0.89–1.36) |
| AP: Attributable proportion, CI: Confidence interval, GS: Grip strength, RERI: Relative excess risk due to interaction, S: Synergy index, T2D: Type 2 diabetes, WC: Waist circumference  Reference group: Low WC / High GS | | | | | | |

| **Table S7. Additive interaction between WC and GS in relation to T2D risk, stratified by age** | | | | | | |
| --- | --- | --- | --- | --- | --- | --- |
|  | **<60 Years** | | | **≥60 Years** | | |
|  | **RERI**  **(95% CI)** | **AP**  **(95% CI)** | **S**  **(95% CI)** | **RERI**  **(95% CI)** | **AP**  **(95% CI)** | **S**  **(95% CI)** |
| High WC x  Low GS | 1.95  (1.48–2.42) | 0.19  (0.15–0.23) | 1.27  (1.20–1.35) | 0.65  (0.38–0.92) | 0.11  (0.06–0.15) | 1.15  (1.08–1.22) |
| Intermediate WC x Low GS | 0.60  (0.31–0.89) | 0.17  (0.09–0.25) | 1.31  (1.12–1.50) | 0.24  (0.04–0.45) | 0.09  (0.02–0.17) | 1.17  (1.01–1.33) |
| High WC x  Intermediate GS | 0.47  (0.03–0.92) | 0.06  (0.00–0.11) | 1.07  (1.00–1.14) | 0.09  (−0.18-0.35) | 0.02  (−0.04-0.07) | 1.02  (0.95–1.09) |
| Intermediate WC x Intermediate GS | 0.25  (−0.03–0.52) | 0.09  (−0.01–0.20) | 1.18  (0.95–1.40) | 0.30  (0.11–0.49) | 0.13  (0.05–0.21) | 1.29  (1.06–1.53) |
| AP: Attributable proportion, CI: Confidence interval, GS: Grip strength, RERI: Relative excess risk due to interaction, S: Synergy index, T2D: Type 2 diabetes, WC: Waist circumference  Reference group: Low WC / High GS | | | | | | |

| **Table S8. Fully adjusted HRs and 95% CIs for the combination of WC and GS in relation to T2D** | | | | |
| --- | --- | --- | --- | --- |
|  | **Cases** | **Person-years** | **HR** | **95% CI** |
| **Low WC** | | | | |
| High GS | 1,170 | 919,992 | 1.00 | – |
| Intermediate GS | 1,426 | 971,874 | 1.04 | 0.97–1.13 |
| Low GS | 2,056 | 910,782 | 1.39 | 1.30–1.50 |
| **Intermediate WC** | | | | |
| High GS | 1,804 | 583,833 | 2.09 | 1.94–2.25 |
| Intermediate GS | 2,117 | 555,312 | 2.37 | 2.20–2.54 |
| Low GS | 2,613 | 525,175 | 2.75 | 2.56–2.94 |
| **High WC** | | | | |
| High GS | 5,551 | 623,032 | 5.82 | 5.46–6.20 |
| Intermediate GS | 5,663 | 574,167 | 5.87 | 5.51–6.26 |
| Low GS | 7,840 | 614,333 | 6.86 | 6.44–7.30 |
| CI: Confidence interval, GS: Grip strength, HR: Hazard ratio, T2D: Type 2 diabetes, WC: Waist circumference  Models were stratified by study region, sex and age group and adjusted for education, socioeconomic status, smoking, alcohol, healthy diet score, sedentary behavior, physical activity, height and family history of diabetes. | | | | |

| **Table S9. HRs and 95% CIs for the combination of WC and NGS in relation to T2D** | | | | |
| --- | --- | --- | --- | --- |
|  | **Cases** | **Person-years** | **HR** | **95% CI** |
| **Low WC** | | | | |
| High NGS | 1,379 | 959,592 | 1.00 | – |
| Intermediate NGS | 1,484 | 958,617 | 1.06 | 0.98–1.14 |
| Low NGS | 1,775 | 882,952 | 1.30 | 1.21–1.40 |
| **Intermediate WC** | | | | |
| High NGS | 1,960 | 571,473 | 2.12 | 1.98–2.27 |
| Intermediate NGS | 2,150 | 558,170 | 2.32 | 2.17–2.48 |
| Low NGS | 2,413 | 533,251 | 2.61 | 2.44–2.78 |
| **High WC** | | | | |
| High NGS | 5,562 | 594,415 | 5.69 | 5.36–6.04 |
| Intermediate NGS | 5,783 | 580,018 | 5.85 | 5.52–6.21 |
| Low NGS | 7,651 | 634,916 | 6.70 | 6.32–7.10 |
| CI: Confidence interval, HR: Hazard ratio, NGS: Normalized grip strength (absolute grip strength in kilograms divided by height in meters squared), T2D: Type 2 diabetes, WC: Waist circumference  Models were stratified by study region, sex and age group and adjusted for education, socioeconomic status, smoking, alcohol, healthy diet score, and sedentary behavior. | | | | |

| **Table S10. HRs and 95% CIs for the combination of WC and GS (Tomkinson cut-offs) in relation to T2D** | | | | |
| --- | --- | --- | --- | --- |
|  | **Cases** | **Person-years** | **HR** | **95% CI** |
| **Low WC** | | | | |
| High GS | 647 | 574,457 | 1.00 | – |
| Intermediate GS | 2545 | 1,593,934 | 1.26 | 1.16–1.38 |
| Low GS | 1460 | 634,257 | 1.75 | 1.60–1.92 |
| **Intermediate WC** | | | | |
| High GS | 1050 | 369,266 | 2.18 | 1.98–2.41 |
| Intermediate GS | 3598 | 919,521 | 2.74 | 2.52–2.98 |
| Low GS | 1886 | 375,533 | 3.46 | 3.17–3.79 |
| **High WC** | | | | |
| High GS | 3332 | 394,030 | 6.36 | 5.85–6.92 |
| Intermediate GS | 9745 | 955,239 | 6.97 | 6.43–7.55 |
| Low GS | 5977 | 462,263 | 8.74 | 8.05–9.49 |
| CI: Confidence interval, GS: Grip strength, HR: Hazard ratio, T2D: Type 2 diabetes, WC: Waist circumference  Models were stratified by study region, sex and age group and adjusted for education, socioeconomic status, smoking, alcohol, healthy diet score, and sedentary behavior. GS categories based on Tomkinson p25/p75 (interpolated from p20/p30 and p70/p80). | | | | |

| **Table S11. Comparison of HRs for incident T2D across different approaches to handling missing covariate data** | | | |
| --- | --- | --- | --- |
|  | **Complete case** | **Multiple imputation** | **Original (Missing Indicator)** |
| **Low WC** | | | |
| High GS | 1.00 (–) | 1.00 (–) | 1.00 (–) |
| Intermediate GS | 1.08 (1.00–1.17) | 1.09 (1.01–1.18) | 1.09 (1.01–1.18) |
| Low GS | 1.53 (1.42–1.65) | 1.54 (1.44–1.66) | 1.54 (1.43–1.66) |
| **Intermediate WC** | | | |
| High GS | 2.13 (1.97–2.29) | 2.12 (1.97–2.28) | 2.12 (1.97–2.28) |
| Intermediate GS | 2.50 (2.33–2.69) | 2.51 (2.34–2.69) | 2.50 (2.33–2.69) |
| Low GS | 3.06 (2.85–3.28) | 3.06 (2.85–3.28) | 3.05 (2.85–3.27) |
| **High WC** | | | |
| High GS | 5.99 (5.61–6.38) | 5.99 (5.62–6.38) | 5.98 (5.61–6.37) |
| Intermediate GS | 6.32 (5.92–6.74) | 6.29 (5.90–6.70) | 6.28 (5.89–6.69) |
| Low GS | 7.72 (7.25–8.23) | 7.71 (7.24–8.20) | 7.68 (7.22–8.17) |
| GS: Grip strength, HR: Hazard ratio, T2D: Type 2 diabetes, WC: Waist circumference, Reference group: Low WC / High GS  HRs were estimated using three approaches to handle missing covariate data: complete case analysis, multiple imputation (10 datasets, 5 iterations each) and missing indicator method. | | | |

**Figure S1. Flow chart of participant inclusion and exclusion**

Participants with missing values in grip strength

N = 551

Participants with pre-existing T2D

N = 9,281

Participants with T2D in the first two years of follow-up

N = 4,154

Baseline population

N = 499,705

Participants with missing values in waist circumference

N = 2,141

Population for analysis

N = 483,578

**Figure S2. Directed acyclic graph**

**
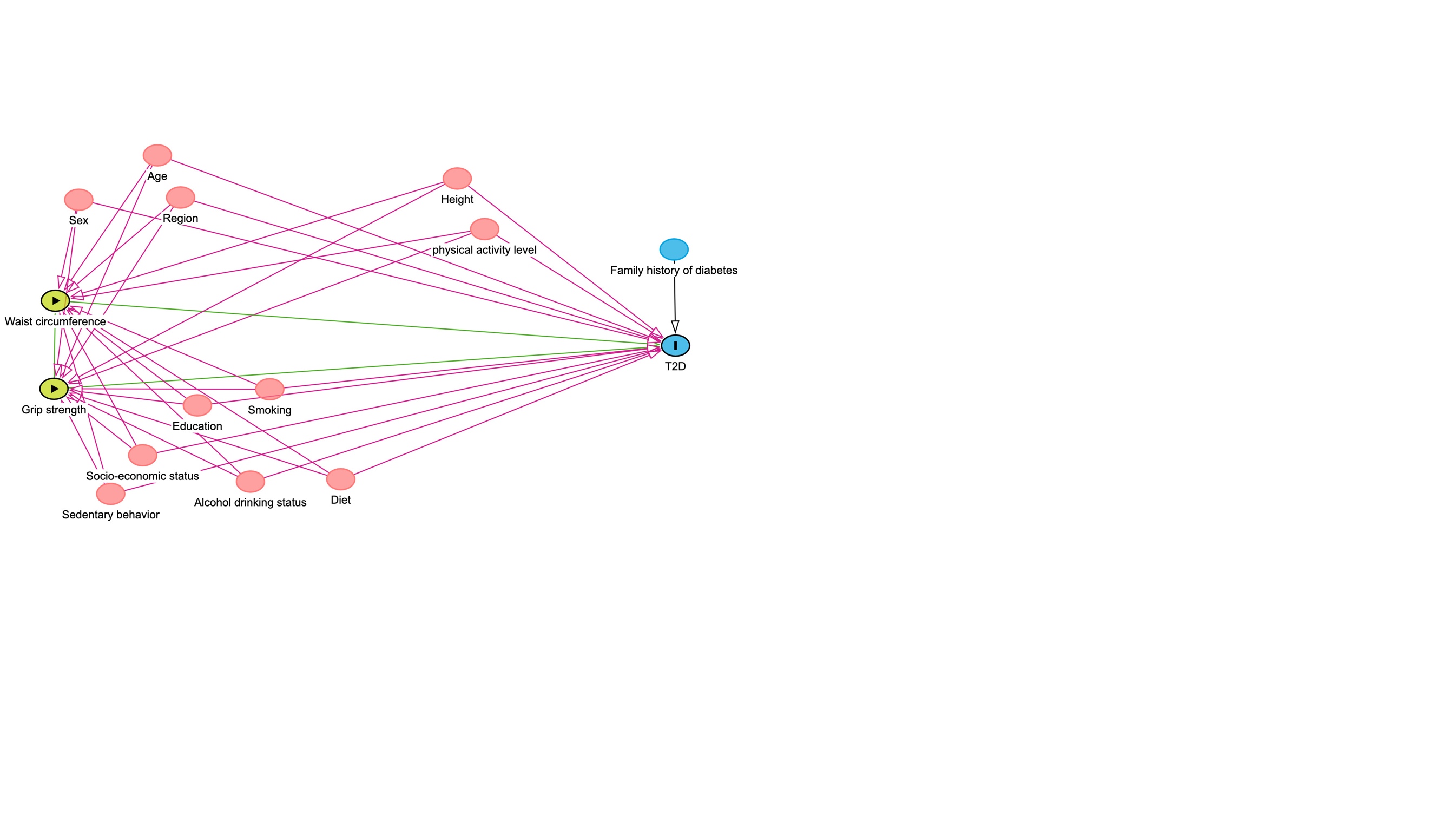
**

Red: Ancestor of exposure and outcome, i.e., a confounder. Blue ancestor of outcome; these are only causes of the outcome, hence, not confounding variables, but adjusting for such variables tends to increase the power of statistical tests [27].


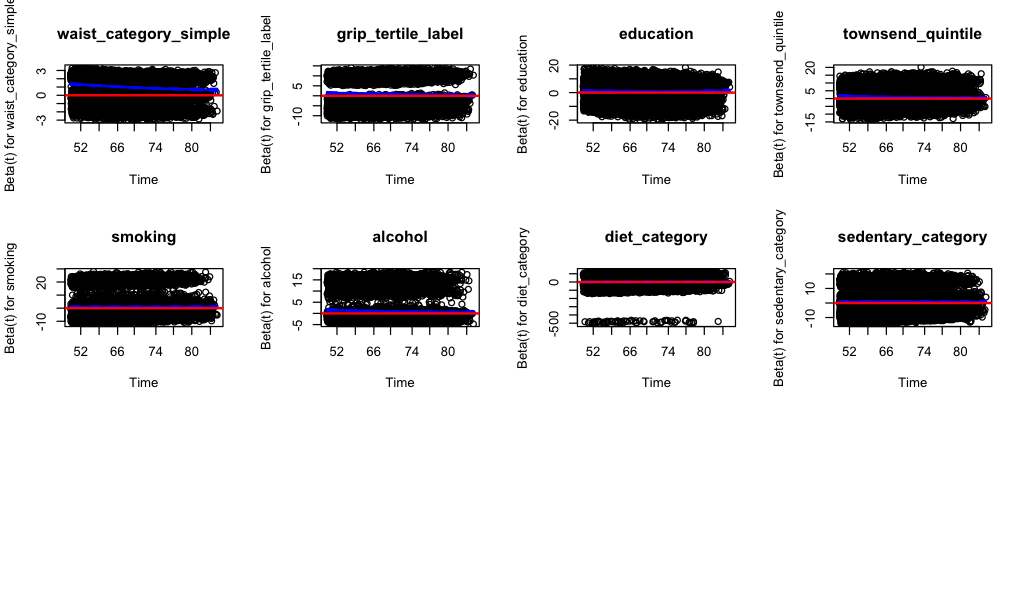
**Figure S3. Scaled Schoenfeld residual plots for separate WC and GS models**


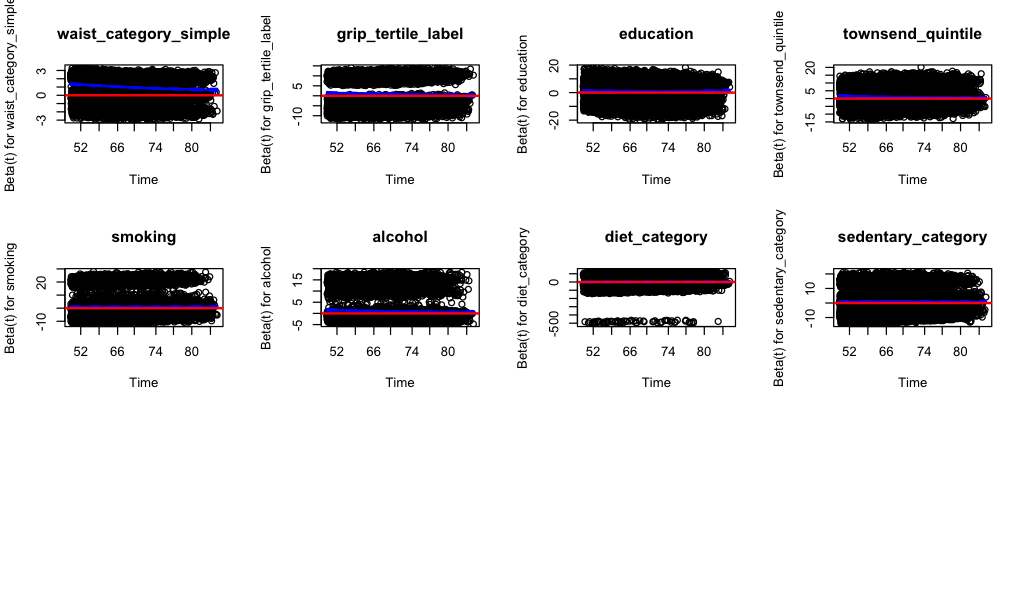


GS: Grip strength, T2D: Type 2 diabetes, WC: Waist circumference

Scaled Schoenfeld residuals plotted against time for the Cox model examining WC and GS as separate (mutually adjusted) exposures with T2D (Table S10, Model 4). The blue line shows the smoothed trend; the solid red line indicates no time-varying effect (beta = 0). Non-horizontal trends indicate time-varying coefficients. Models stratified by sex, age group, and region; adjusted for education, socioeconomic status, smoking, alcohol, diet quality, and sedentary behavior.

**Figure S4. Scaled Schoenfeld residual plots for the joint exposure model**


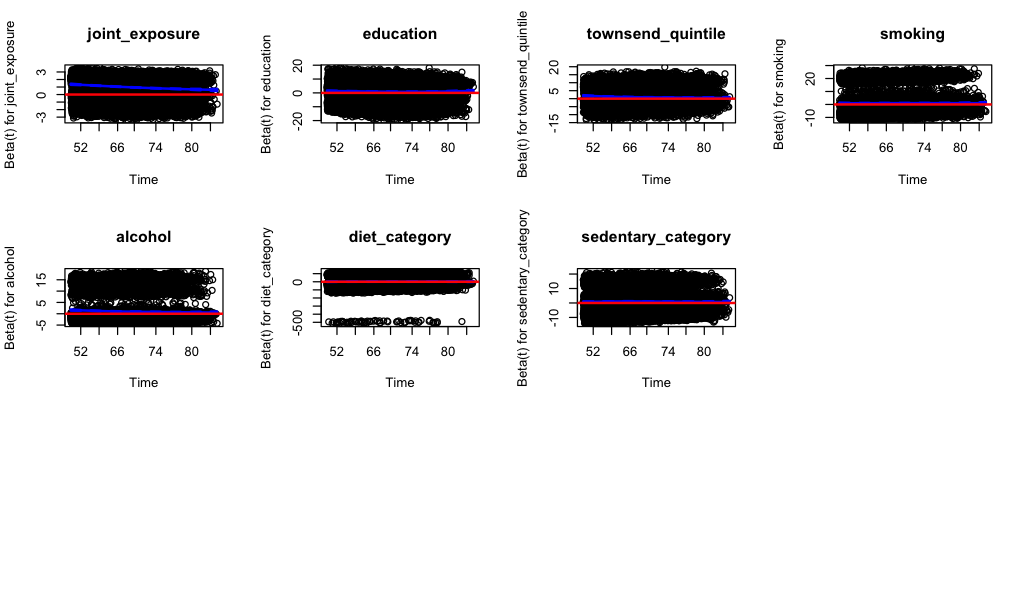


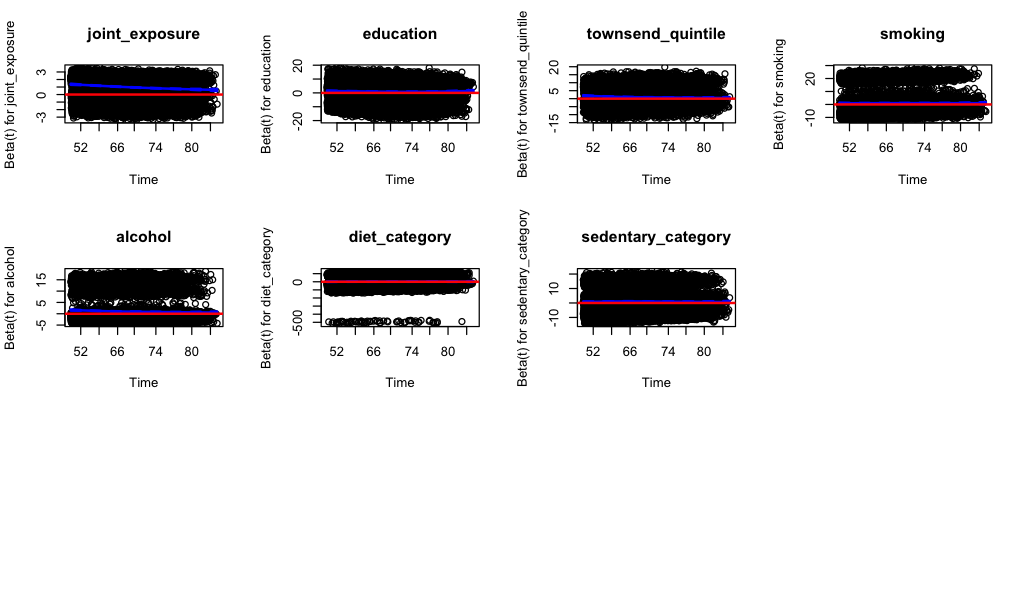


GS: Grip strength, T2D: Type 2 diabetes, WC: Waist circumference

Scaled Schoenfeld residuals plotted against time for the Cox model examining joint associations of WC and GS with T2D (Table S10, Model 2). The blue line shows the smoothed trend; the solid red line indicates no time-varying effect (beta = 0). Non-horizontal trends indicate time-varying coefficients. Model stratified by sex, age group, and region; adjusted for education, socioeconomic status, smoking, alcohol, diet quality, and sedentary behavior.

**Figure S5. Non-linear association between WC and T2D risk stratified by GS tertiles**

**
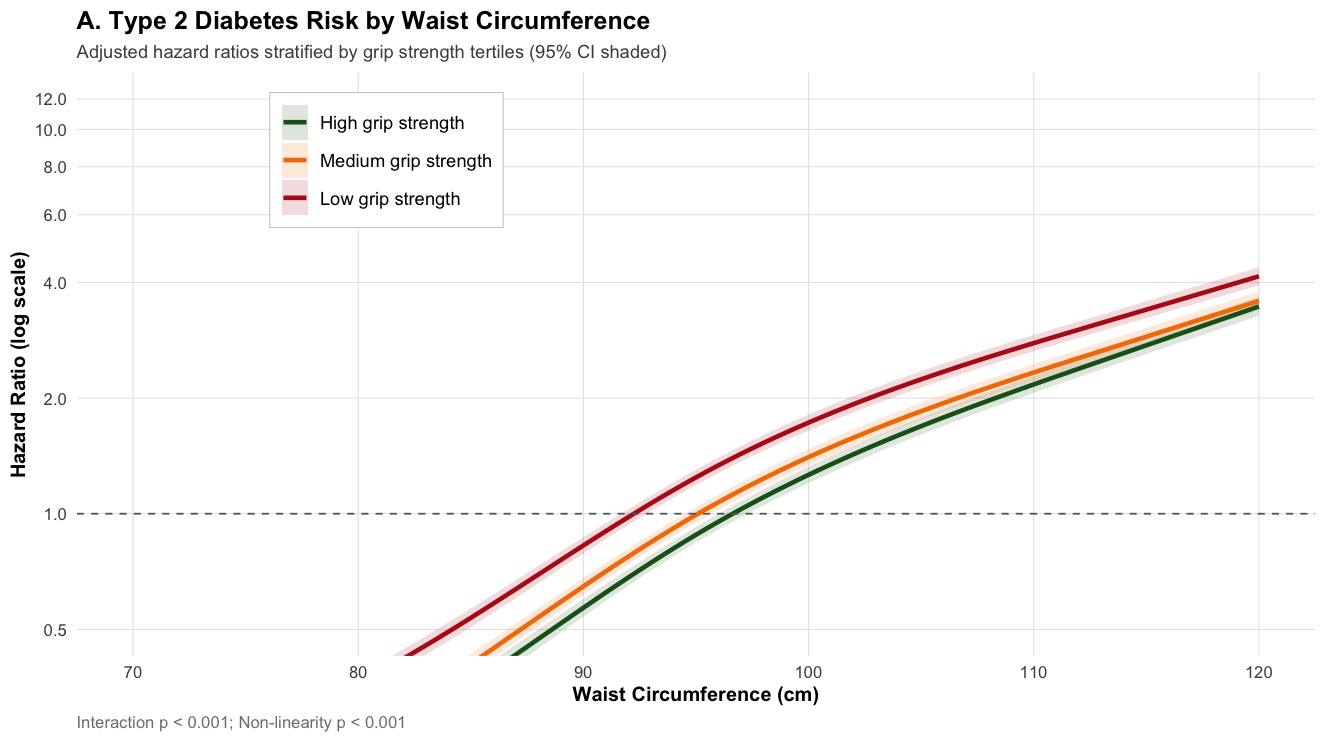
**

CI: Confidence interval, GS: Grip strength, HR: Hazard ratio, T2D: Type 2 diabetes, WC: Waist circumference

HRs are shown on a logarithmic scale. Shaded areas represent 95% CIs. The dashed horizontal line at HR = 1.0 represents no association. WC was modeled using restricted cubic splines with 4 knots. GS were categorized in age- and sex-specific tertiles (Table S1). Models were stratified by sex, region, and age group, and adjusted for education, socioeconomic status, smoking status, alcohol consumption, diet quality, and sedentary behavior.

**Figure S6. Association between GS and T2D risk stratified by WC categories**


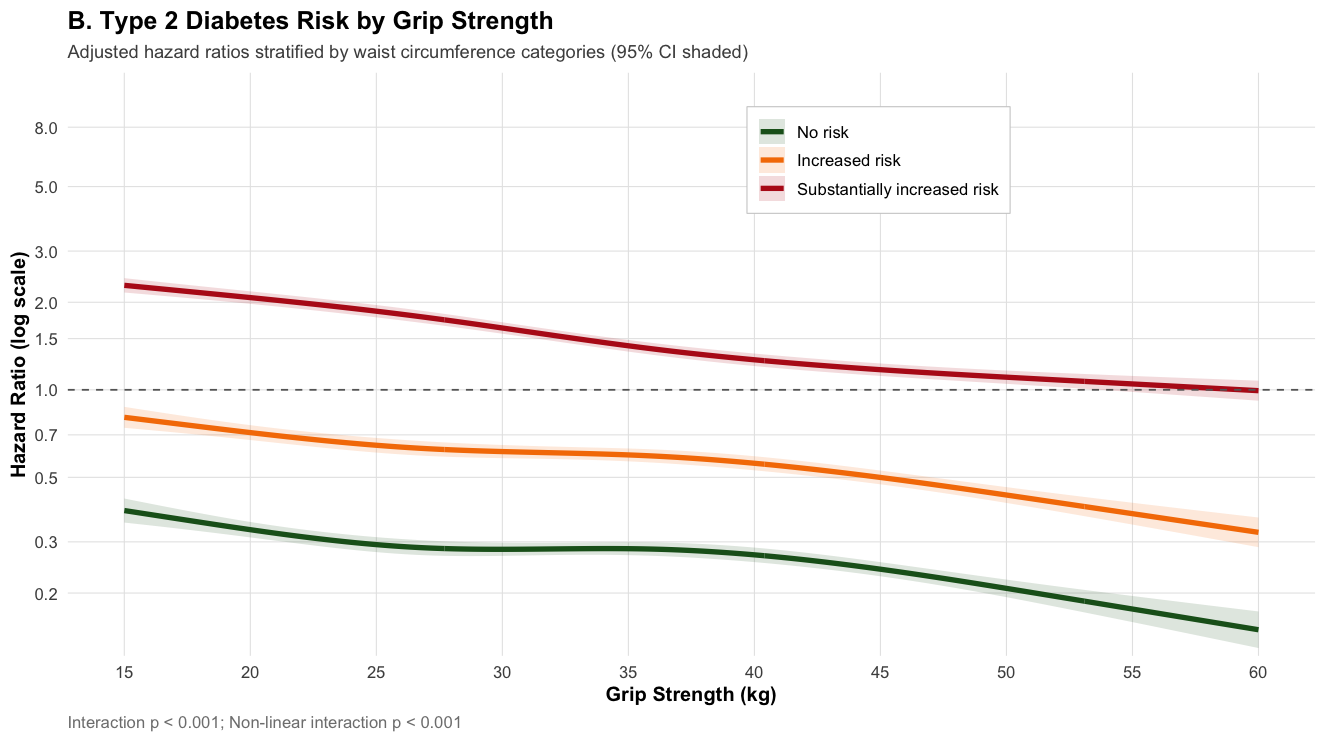


CI: Confidence interval, GS: Grip strength, HR: Hazard ratio, T2D: Type 2 diabetes, WC: Waist circumference

HRs are shown on a logarithmic scale. Shaded areas represent 95% CIs. The dashed horizontal line at HR = 1.0 represents no association. GS was modeled using restricted cubic splines with 4 knots. WC risk categories: No risk: ≤94/80 cm for men/ women; Increased risk: 94–102 cm/ 80–88 cm; Substantially increased risk: >102/ >88 cm. Models were stratified by sex, region, and age group, and adjusted for education, socioeconomic status, smoking status, alcohol consumption, diet quality, and sedentary behavior.
