# Supplementary material for: Waist circumference and grip strength and their joint relations to type 2 diabetes incidence in UK Biobank
Source: BMC Med. 2026 May 7;24:299. doi: 10.1186/s12916-026-04907-8 (PMC13154465; doi:10.1186/s12916-026-04907-8)
Supplement: Supplementary file 2 — Additional file 2 STROBE Statement [file 12916_2026_4907_MOESM2_ESM.pdf]

STROBE Statement—checklist of items that should be included in reports of observational studies

	Item No.	Recommendation	Page No.	Relevant text from manuscript
**Title and abstract**	1	(*a*) Indicate the study's design with a commonly used term in the title or the abstract	1-2	We examined the separate and joint associations of waist circumference and grip strength with incident T2D among 483,578 adults aged 40–69 years (55% women) without T2D at baseline (2006–2010) from UK Biobank.
		(*b*) Provide in the abstract an informative and balanced summary of what was done and what was found	1-2	We examined the separate and joint associations of waist circumference and grip strength with incident T2D among 483,578 adults aged 40–69 years. Joint analyses showed the highest risk among participants with the combination of high waist circumference and low grip strength (HR 7.68, 95% CI 7.22–8.17)
**Introduction**				
Background/rationale	2	Explain the scientific background and rationale for the investigation being reported	3	Despite the growing body of evidence on the separate roles of abdominal obesity and muscular fitness, their combined influence on T2D risk remains underexplored; prospective data integrating abdominal obesity with handgrip strength are limited to a single Asian study which reported inconclusive results for the joint association. No prior study has examined this joint association in a European population.
Objectives	3	State specific objectives, including any prespecified hypotheses	3	The present study investigated the separate and joint associations of waist circumference and grip strength with incident T2D in a large cohort of adults in UK Biobank.
**Methods**				
Study design	4	Present key elements of study design early in the paper	3	UK Biobank is a prospective cohort that recruited over 500,000 UK participants aged 40-69 years at baseline (2006-2010).
Setting	5	Describe the setting, locations, and relevant dates, including periods of recruitment, exposure, follow-up, and data collection	3-4	UK Biobank is a prospective cohort that recruited over 500,000 UK participants aged 40-69 years at baseline (2006-2010). Follow-up began at the assessment date and ended at the date of T2D diagnosis, date of complete follow-up (October 2022 for England, August 2022 for Scotland and May 2022 for Wales), loss to follow-up, or date of death, whichever occurred first
Participants	6	(*a*) *Cohort study*—Give the eligibility criteria, and the sources and methods of selection of participants. Describe methods of follow-up<br>*Case-control study*—Give the eligibility criteria, and the sources and methods of case ascertainment and control selection.	3-4	We excluded participants with prevalent T2D – any individual with hospital inpatient records of T2D on or before baseline – and those with missing values for any exposure or outcome variable. To mitigate potential reverse causation, we excluded T2D cases that occurred within the first two years of follow-up, resulting in a final study population of 483,578 individuals."<br><br>Participants' vital status was determined through linkage with routine healthcare data and national death registries.

		Give the rationale for the choice of cases and controls<br>*Cross-sectional study*—Give the eligibility criteria, and the sources and methods of selection of participants		
		(*b*) *Cohort study*—For matched studies, give matching criteria and number of exposed and unexposed<br>*Case-control study*—For matched studies, give matching criteria and the number of controls per case		
Variables	7	Clearly define all outcomes, exposures, predictors, potential confounders, and effect modifiers. Give diagnostic criteria, if applicable	4-5	**Exposure:** Waist circumference was categorized according to World Health Organization sex-specific thresholds into three waist circumference groups. Grip strength was assessed using a Jamar hydraulic hand dynamometer following a standardized protocol.<br><br>**Outcome:** The endpoint was incident T2D, identified through linkage with hospital inpatient records of ICD-10 code E11 or equivalent ICD-9 codes (e.g., 250.00).<br><br>**Confounders:** We stratified by sex, age, and study region, and adjusted for smoking, alcohol use, socio-economic status, education, sedentary behavior, and healthy diet score.
Data sources/ measurement	8*	For each variable of interest, give sources of data and details of methods of assessment (measurement). Describe comparability of assessment methods if there is more than one group	4	**Waist circumference:** Clinical staff measured waist circumference in centimeters using a Seca 200cm tape measure at the smallest part of the trunk, or the belly button, during exhalation.<br><br>**Grip strength:** Grip strength was assessed using a Jamar hydraulic hand dynamometer following a standardized protocol. Participants performed two maximal grip strength attempts per hand, and the highest value was retained.<br><br>**Outcome:** Incident T2D was identified through linkage with hospital inpatient records of ICD-10 code E11 or equivalent ICD-9 codes, with the incident date assigned as the first diagnosis.<br><br>**Covariates:** Potential confounding covariates were determined using evidence-based directed acyclic graphs (DAGs), with details in Additional file 1: Figure S2.
Bias	9	Describe any efforts to address potential sources of bias	3, 5	To mitigate potential reverse causation, we excluded T2D cases that occurred within the first two years of follow-up.

				In sensitivity analyses, we additionally adjusted for physical activity volume, height, and family history of diabetes to evaluate the robustness of the results with respect to further potential confounding.
Study size	10	Explain how the study size was arrived at	3	We excluded participants with prevalent T2D – any individual with hospital inpatient records of T2D on or before baseline – and those with missing values for any exposure or outcome variable. To mitigate potential reverse causation, we excluded T2D cases that occurred within the first two years of follow-up, resulting in a final study population of 483,578 individuals (Additional file 1: Figure S1).

Continued on next page

Quantitative variables	11	Explain how quantitative variables were handled in the analyses. If applicable, describe which groupings were chosen and why	4	Waist circumference was categorized according to World Health Organization sex-specific thresholds into three waist circumference groups: substantially increased risk (high: >102 cm for men, >88 cm for women), increased risk (intermediate: 95–102 cm for men, 81–88 cm for women); no risk (low: ≤94 cm for men, ≤80 cm for women). Given that UK Biobank participants tend to be healthier than the general population, we used age- and sex-specific tertiles to categorize grip strength into 'low', 'intermediate' and 'high' to better reflect the distribution within this cohort rather than applying recently proposed reference values.
Statistical methods	12	(a) Describe all statistical methods, including those used to control for confounding	5	We performed Cox proportional hazards regression using age as the underlying time scale to estimate hazard ratios (HRs) and corresponding 95% confidence intervals (CIs) for waist circumference and grip strength in mutually adjusted models. We stratified by sex, age, and study region, and adjusted for smoking, alcohol use, socio-economic status, education, sedentary behavior, and healthy diet score.
		(b) Describe any methods used to examine subgroups and interactions	5	Further analyses were stratified by sex and by age (<60 years, ≥60 years) to assess patterns across major characteristics. We tested for multiplicative interaction by generating cross-product terms between waist circumference and grip strength, the statistical significance of which were tested using a Likelihood ratio test. We additionally evaluated additive interaction using the relative excess risk due to interaction (RERI), the attributable proportion (AP), and the synergy index (S).
		(c) Explain how missing data were addressed	6	Missing values in covariates were handled using missing indicator categories in the primary analyses. To assess the influence of missing values, we conducted multiple imputation using chained equations (10 datasets with 5 iterations each).
		(d) Cohort study—If applicable, explain how loss to follow-up was addressed<br>Case-control study—If applicable, explain how matching of cases and controls was addressed<br>Cross-sectional study—If applicable, describe analytical methods taking account of sampling strategy	4	Completeness of follow-up was ensured by additional linkage with national health registries, which provide near-complete case ascertainment and accurate survival time.
		(e) Describe any sensitivity analyses	5, 6	In sensitivity analyses, we additionally adjusted for physical activity volume, height, and family history of diabetes to evaluate the robustness of the results with respect to further potential confounding. We applied normative reference values for absolute grip strength proposed by Tomkinson et al., classifying participants within each age-sex stratum as having low (<25th percentile), intermediate (25th-75th percentile), or high (>75th percentile) strength. To assess the influence of missing values, we conducted multiple imputation using chained equations (10 datasets with 5 iterations each)

**Results**				
Participants	13*	(a) Report numbers of individuals at each stage of study—eg numbers potentially eligible, examined for eligibility, confirmed eligible, included in the study, completing follow-up, and analysed	3, Additional file 1: 9	resulting in a final study population of 483,578 individuals<br>Additional file 1: Figure S1
		(b) Give reasons for non-participation at each stage	Additional file 1: 9	Additional file 1: Figure S1
		(c) Consider use of a flow diagram	Additional file 1: 9	Additional file 1: Figure S1
Descriptive data	14*	(a) Give characteristics of study participants (eg demographic, clinical, social) and information on exposures and potential confounders	6, 7, 21, 22	Our analytical cohort included individuals with a mean age of 56.4 ± 8.1 years at baseline, of whom 55% were women. Participants with a low waist circumference and higher grip strength were younger, had higher educational attainment and socioeconomic status, were less likely to smoke, consumed a higher-quality diet, were more likely to drink alcohol and had less sedentary time.<br><br>Tables 1a and 1b
		(b) Indicate number of participants with missing data for each variable of interest	Additional file 1: 9	Additional file 1: Figure S1
		(c) Cohort study—Summarise follow-up time (eg, average and total amount)	7	Over a follow-up of 13.0 years (totaling 6,278,500 person-years), 30,240 participants (6.3%) developed T2D.
Outcome data	15*	Cohort study—Report numbers of outcome events or summary measures over time	7	Over a follow-up of 13.0 years (totaling 6,278,500 person-years), 30,240 participants (6.3%) developed T2D.
		Case-control study—Report numbers in each exposure category, or summary measures of exposure		
		Cross-sectional study—Report numbers of outcome events or summary measures		
Main results	16	(a) Give unadjusted estimates and, if applicable, confounder-adjusted estimates and their precision (eg, 95% confidence interval). Make clear which confounders	1, 2, 8	Unadjusted estimates are not reported.<br><br>Compared to individuals with low waist circumference, HRs were 2.11 (95% CI 2.03–2.19) for those with intermediate and 5.48 (95% CI 5.30–5.66) for those with high waist circumference.

	were adjusted for and why they were included		Compared to individuals with high grip strength, HRs were 1.08 (95% CI 1.05–1.11) for those with intermediate and 1.35 (95% CI 1.32–1.39) for those with low grip strength. Joint analyses showed the highest risk among participants with the combination of high waist circumference and low grip strength (HR 7.68, 95% CI 7.22–8.17). Models were stratified by study region, sex and age group, and adjusted for education, socioeconomic status, smoking, alcohol, healthy diet score and sedentary behavior.
	(b) Report category boundaries when continuous variables were categorized	8, Additional file 1: 1	Waist circumference: substantially increased risk (high: >102 cm for men, >88 cm for women), increased risk (intermediate: 95–102 cm for men, 81–88 cm for women); no risk (low: ≤94 cm for men, ≤80 cm for women). We used age- and sex-specific tertiles to categorize grip strength into 'low', 'intermediate' and 'high' (Additional file 1: Table S1).
	(c) If relevant, consider translating estimates of relative risk into absolute risk for a meaningful time period		Not reported.

Continued on next page

Other analyses	17	Report other analyses done—eg analyses of subgroups and interactions, and sensitivity analyses	8, 9	The association of high waist circumference and low grip strength with T2D was stronger in women (HR 10.24; 95% CI 9.16–11.46) than in men (HR 6.47; 95% CI 6.00–6.98). The association was stronger in participants younger than 60 years (HR 10.12; 95% CI 9.17–11.17) than in those aged 60 years or older (HR 6.04; 95% CI 5.57–6.54). After additional adjustment for physical activity, height, and family history of diabetes, individuals with the combination of high waist circumference and low grip strength had an almost sevenfold higher hazard of developing T2D (HR: 6.86; 95% CI 6.44–7.30). Applying alternative age- and sex-specific cut-offs for normalized grip strength yielded similar patterns, with HRs ranging from 5.69 to 6.70 in the high waist circumference group.
**Discussion**				
Key results	18	Summarise key results with reference to study objectives	10	Higher waist circumference and lower handgrip strength were each separately associated with increased diabetes risk. By demonstrating excess risk on the additive scale among individuals with both high waist circumference and low handgrip strength, our findings identify a distinct high-risk phenotype characterized by the coexistence of elevated metabolic load and reduced metabolic reserve.
Limitations	19	Discuss limitations of the study, taking into account sources of potential bias or imprecision. Discuss both direction and magnitude of any potential bias	13, 14	Grip strength and waist circumference were measured only once at baseline, which precluded assessment of temporal changes or training-induced improvements during follow-up. Residual confounding from unmeasured lifestyle or genetic factors cannot be fully excluded. Formal tests of the proportional hazards assumption indicated statistical departures. Our HRs should therefore be interpreted as weighted temporal averages across the follow-up period. Our observational design precludes causal inference. Generalizability is limited by the volunteer composition of the UK Biobank cohort, which is healthier and less socioeconomically diverse than the general population.
Interpretation	20	Give a cautious overall interpretation of results considering objectives, limitations, multiplicity of analyses, results from similar studies, and other relevant evidence	10, 12, 13,	This is the first prospective study in a large European cohort to jointly examine abdominal obesity and handgrip strength in relation to incident diabetes. The more-than-additive joint association observed in our study raises the possibility that among individuals with excess abdominal adiposity and low muscular strength, jointly addressing both factors may confer greater benefit than targeting either alone, although this hypothesis requires confirmation in intervention studies and should be interpreted cautiously given the modest magnitude of the additive excess risk. Routine joint assessment of waist circumference and handgrip strength is appealing given that both measures are low-cost and scalable, however, formal evaluation of their incremental predictive utility beyond established risk factors is needed before recommending their combined use for clinical risk stratification. Research on the combined role of abdominal adiposity and handgrip strength in diabetes risk is limited to one Asian prospective study and one multicountry cross-sectional study. Both identified abdominal obesity as the key factor associated with diabetes, with similar risk estimates for abdominal obesity alone and for the combined phenotype.

Generalisability	21	Discuss the generalisability (external validity) of the study results	14	Generalizability is limited by the volunteer composition of the UK Biobank cohort, which is healthier and less socioeconomically diverse than the general population.
**Other information**				
Funding	22	Give the source of funding and the role of the funders for the present study and, if applicable, for the original study on which the present article is based	15, 16	Funding for IIG_FULL_2021_027 was obtained from World Cancer Research Fund (WCRF UK), as part of the World Cancer Research Fund International grant programme. This study was supported by the German Research Foundation (BA 5459/2-1). The research was designed, conducted, analysed, and interpreted by the authors entirely independently of these funding sources. The funder had no role in study design, data acquisition and analysis, decision to publish, or preparation of the manuscript.

*Give information separately for cases and controls in case-control studies and, if applicable, for exposed and unexposed groups in cohort and cross-sectional studies.

Note: An Explanation and Elaboration article discusses each checklist item and gives methodological background and published examples of transparent reporting. The STROBE checklist is best used in conjunction with this article (freely available on the Web sites of PLoS Medicine at http://www.plosmedicine.org/, Annals of Internal Medicine at http://www.annals.org/, and Epidemiology at http://www.epidem.com/). Information on the STROBE Initiative is available at www.strobe-statement.org.
